# Climate Change Drives Bathymetric Shifts in Taxonomic and Trait Diversity of Deep‐Sea Benthic Communities

**DOI:** 10.1111/gcb.70407

**Published:** 2025-08-05

**Authors:** M. Rakka, A. Metaxas, M. Nizinski

**Affiliations:** ^1^ Department of Oceanography Dalhousie University Halifax Nova Scotia Canada; ^2^ NOAA Fisheries, Office of Science and Technology, National Systematics Laboratory Smithsonian Institution Washington DC USA

**Keywords:** benthic communities, community ecology, deep sea, deep‐sea corals, functional ecology, joint species distribution models, ocean warming

## Abstract

Climate‐induced changes in environmental gradients can cause shifts in ranges of organisms and community composition, with concomitant effects on ecosystem functions. Throughout geological time, deeper depths have been highlighted as refugia for biodiversity and ecosystem functions under a warming climate. Although the deep ocean provides several important ecosystem services, contemporary research on climate effects at the community and ecosystem levels has been limited to the upper 200 m of the water column. As a result, our knowledge of climate‐induced impacts on the functions of deep‐sea ecosystems is scarce. In this study, we examined climate‐induced changes in deep‐sea communities at a climate‐change hotspot, the Gulf of Maine and adjacent continental slope in the Northwest Atlantic. We focused on deep‐water coral communities, which are among the most diverse in the deep sea. Using a joint species distribution model, we projected and examined community composition, taxonomic diversity, and trait diversity of deep‐water coral communities under two climate scenarios for the end of the century (2100). We found extensive shifts of suitable habitat for several coral genera from 500–1000 to 1500–2000 m, mostly attributed to warming in the upper 1000 m. This led to substantial reduction (30%–60%) in the existing taxonomic and functional richness at the upper continental slope, alongside gains in richness (10%–15%) at the lower continental slope and bathyal zone. Our study is the first to report extensive shifts in biodiversity from mesopelagic to bathyal depths, which will inevitably cause redistribution of ecosystem functions and services. These results showcase that climate change impacts at the ecosystem level are not restricted to shallow depths and highlight that further knowledge of them is essential for efficient conservation, planning, and management.

## Graphical abstract

This study uses modeling techniques to predict the characteristics of deep‐water coral communities under climate scenarios. It reveals that climate change will drive shifts of many deep‐water corals to deeper waters, leading to major changes in biodiversity and ecosystem functions of deep‐water coral communities. The results highlight that climate change is not only affecting shallow waters but is also transforming deep‐sea ecosystems, underscoring the need for further research and marine conservation in these depths.

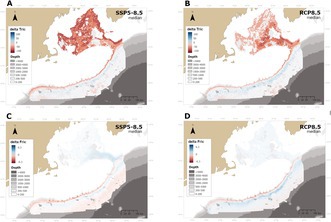

## 1 Introduction

Climate change can have complex effects across all levels of ecological organization, including species, populations, communities, and ecosystems. Although previous studies initially focused on impacts on individual species, a better understanding of impacts at the ecosystem level is essential (Bindoff et al. 2019; IPCC 2021). Ecosystem‐level processes depend largely on local community composition and species interactions. The structure of the local community and the prevailing traits within it define energy flow and trophic interactions, which in turn determine important ecosystem functions such as productivity and nutrient recycling (Gravel et al. 2016; van der Plas 2019). Ecosystem functions also depend on diversity, which can be quantified taxonomically (e.g., species richness) or functionally (functional richness based on traits). Both aspects of diversity are critical for maintaining ecosystem functions and services under disturbance, since high biodiversity can increase the range of potential responses and support resilience (Mace et al. 2012; Oliver et al. 2015). Due to the intrinsic links between community composition, diversity, and ecosystem functions, projecting climate change impacts on community assembly, the process by which local species pools are formed, provides useful insights into impacts on ecosystem functions and services.

Community approaches have been widely used to unravel climate change impacts in the marine realm. By using historical datasets on species occurrences and environmental variables, previous studies have highlighted that environmental change is already causing shifts in species distributions, with concomitant changes in the composition of marine communities (Dulvy et al. 2008; Nye et al. 2009; Kortsch et al. 2015). Warming, in particular, has caused extensive shifts in latitudinal and depth ranges for both pelagic and benthic species, leading to high species turnover and changes in biodiversity patterns worldwide (Hiddink et al. 2015; Pinsky et al. 2020; Worm and Lotze 2021). In similar conditions across geological time, species distributions and biodiversity have shifted extensively towards higher latitudes and deeper depths (Schneider 2018). Inevitably, changes in the spatial distribution of communities will cause spatial shifts in ecosystem functions and services. Biodiversity loss can disrupt species interactions, alter food webs, and modify energy flow (Johnson et al. 2011; Kortsch et al. 2015). High species turnover can have similar consequences, especially when functional diversity and the prevailing traits of the community are affected (Ferrari et al. 2016; Takolander et al. 2017; McGinty et al. 2021). Paleontological research and modern observations on coastal ecosystems have highlighted the role of the deeper ocean as a potential refuge for biodiversity and ecosystem functions under warming climate conditions (Graham et al. 2007; Lauer and Reaka 2022; Schneider 2018). However, apart from paleontological studies, most research on the effects of climate change at the community and ecosystem levels has focused on the upper 200 m of the water column. Our knowledge of these processes in the deep sea, defined here as deeper than 200 m, is extremely limited.

Deep‐sea ecosystems provide several important ecosystem services. They play a fundamental role in biogeochemical cycles, particularly nutrient recycling and carbon sequestration (Thurber et al. 2014), and host rich biodiversity, including many species of economic interest (Ramirez‐Llodra 2020). Among the richest communities in the deep sea are those formed by sessile megainvertebrates, such as corals and sponges (Henry and Roberts 2017; Xavier et al. 2023). These taxa are important ecosystem engineers as they provide habitat and fuel nutrient recycling, often supporting a highly diverse assemblage of associated fauna, including invertebrates and fishes (L. Buhl‐Mortensen and Buhl‐Mortensen 2018; Hanz et al. 2022). Moreover, many of these habitat‐forming species are slow growing and have lifespans of up to hundreds of years (Mortensen and Buhl‐Mortensen 2005; Sherwood and Edinger 2009). Due to their importance and high vulnerability to disturbance, communities of deep‐water corals and sponges have been classified as Vulnerable Marine Ecosystems (VMEs) and are protected by several international and national agreements (e.g., FAO 2009; OSPAR 2010; Wright et al. 2021).

Despite their remoteness, deep‐sea benthic ecosystems are already experiencing climate‐driven environmental shifts, with further climate‐induced pressures expected, including warming, acidification, deoxygenation, as well as changes in ocean circulation and food availability (Sweetman et al. 2017; Kwiatkowski et al. 2020). To date, the potential impacts of such changes on deep‐sea communities and their biodiversity have mostly been studied on communities of fishes (Cartes et al. 2015; Emblemsvåg et al. 2020; Lin et al. 2024) and benthic meiofauna (Yasuhara and Danovaro 2016; Doi et al. 2021). For communities formed by sessile megafauna, climate impacts have been mostly inferred from studies on individual species. More specifically, climate projections using species distribution models have predicted a loss of suitable habitat for several habitat‐forming species, including deep‐sea corals in the North Atlantic (Morato et al. 2020; Gasbarro et al. 2022), the Pacific ocean (Anderson et al. 2022), and the Mediterranean Sea (Georges et al. 2024). Shifts to higher latitudes and deeper depths have also been projected for some deep‐sea corals (Anderson et al. 2022) and sponges (Beazley et al. 2021). While these changes have been mostly attributed to warming, ocean acidification and the shoaling of the aragonite/calcite horizon may limit the bathymetric range shifts for some of these species, particularly for corals, which calcify to build their skeletons (Hennige et al. 2020; Morato et al. 2020). However, current research is restricted to a few species, and empirical, long‐term observations are lacking (Levin 2021).

Recent development of statistical tools in community ecology, such as joint species distribution models, enables the projection of community composition under climate change scenarios, providing opportunities to examine climate change impacts at higher ecological levels (Maguire et al. 2015; D'Amen et al. 2017). These powerful tools have been increasingly used in marine studies to understand predator–prey interactions (Roberts et al. 2023), investigate community assembly rules (Montanyès et al. 2023; Xu et al. 2025) and more recently, to study climate impacts (Lin et al. 2024; Roberts et al. 2022). Their application to deep‐sea benthic ecosystems remains limited, with studies specifically focusing on mapping epibenthic communities to aid spatial management (Murillo et al. 2020; Stephenson et al. 2024). Despite the demonstrated power and flexibility of joint species distribution models, to date, no study has employed them to better understand climate change effects on deep‐sea benthic communities.

We examined the potential impacts of climate change on deep‐water coral communities in the Gulf of Maine (GOM) and the adjacent continental slope in the Northwest Atlantic Ocean. This area is considered one of the fastest changing regions in the world in response to climate, as rapid and extensive environmental changes, particularly warming of coastal and deep waters, have already been documented (Saba et al. 2016; Gonçalves Neto et al. 2021). This region also hosts rich deep‐sea benthic communities, primarily formed by deep‐water corals (Quattrini et al. 2015; L. Buhl‐Mortensen and Buhl‐Mortensen 2018). We used a joint species distribution model previously developed for the area by Rakka et al. (2025) to produce projections for 30 deep‐water coral genera under future climate scenarios for the end of the century (2100). We obtained environmental projections from two sources: a global database that includes the latest predictions from the Sixth Phase of the Coupled Model Intercomparison Project (IPCC 2021) and a regional oceanographic model with better resolution of the complex circulation patterns in the area, particularly the effect of the Gulf Stream (Alexander et al. 2020). We further analyze model predictions by using metrics of biodiversity and trait diversity to better understand prospective changes in coral communities and their function. In addition, we report model uncertainties based on the two environmental projections. To our knowledge, this is the first study to address climate change impacts on these important benthic communities beyond the species level. As the deep sea provides several critical ecosystem services that are intrinsically linked to global systems, this knowledge is essential to fully understand climate impacts on the marine realm.

## 2 Materials and Methods

### 2.1 Study Area

**FIGURE 1:**
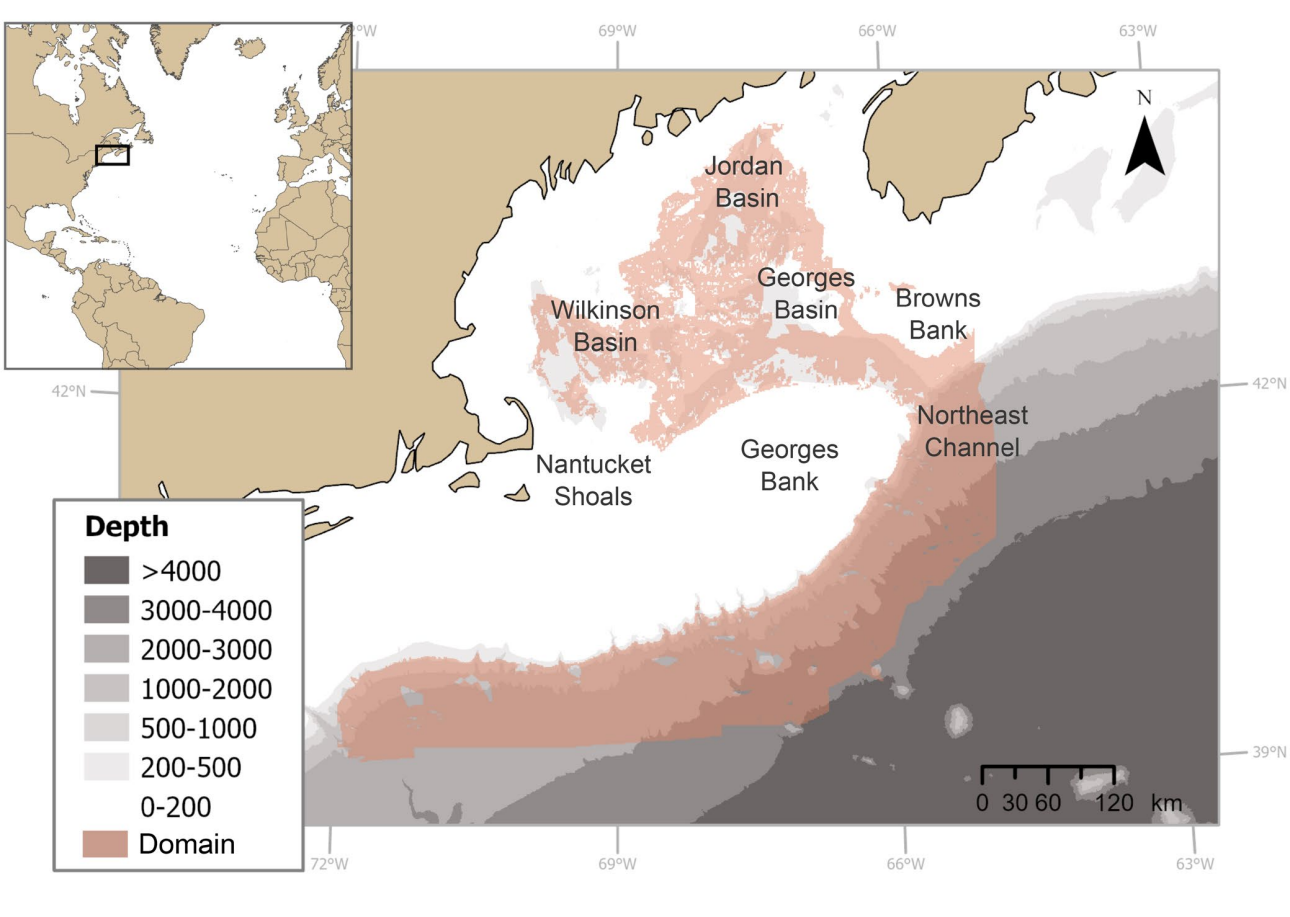
Study area and model domain (pink shaded area) of the constructed Hierarchical Model of Species Communities (HMSC). Map lines delineate study areas and do not necessarily depict accepted national boundaries.

Our study area included the continental shelf and slope off New England (USA) and Nova Scotia (Canada) (Figure 1), which hosts a rich diversity of deep‐water corals, including scleractinian, octocoral, and antipatharian species (Kelly et al. 2010; Auster et al. 2013; Quattrini et al. 2015). The hydrodynamic regime in the area is the result of complex interactions among several oceanographic components. Cold, fresh waters of subarctic origin dominate on the continental shelf, while slope waters are influenced by the Labrador Current that brings cold, fresh water from the north and the Gulf Stream that transports warm, salty water from the south (Townsend et al. 2015; New et al. 2021). Due to the interactions among these components, the continental shelf and slope are characterized by extreme diurnal, annual, and decadal variability (Du et al. 2021). During the past decades, warming has been extensively recorded in the area, often attributed to direct intrusions of the Gulf Stream onto the continental shelf and slope (Du et al. 2021; Gonçalves Neto et al. 2021; Balch et al. 2022). Climate projections predict further warming in the upcoming years, leading to the characterization of this area as a climate change hotspot (Alexander et al. 2020; Brickman et al. 2021).

### 2.2 Model

We used a Hierarchical Model of Species Communities (HMSC, Ovaskainen et al. 2017) that was previously developed for the same study area by Rakka et al. (2025). This modeling framework allows the integration of environmental data, biological data such as species occurrence or abundance, as well as data on biological traits to answer questions related to the mechanisms of community assembly across space and time. It assumes that species distributions are the result of stochastic and deterministic processes known as assembly mechanisms. The model framework focuses on two deterministic processes, environmental filtering, which refers to the environmental factors that prevent the establishment of species in a certain area, and biotic filtering, which represents species interactions. Environmental filtering is modeled through the incorporation of data on environmental variables and species traits, while biotic interactions are modeled through the inclusion of latent variables (Ovaskainen et al. 2017). Latent variables are also used as random factors to account for dependencies and spatial autocorrelation (Ovaskainen and Abrego 2020). By considering interactions, this method allows the inclusion of less common species, which are generally more challenging to incorporate in distribution models. Building on Rakka et al. (2025), where we applied the HMSC model to provide insights into the processes of community assembly for deep‐water corals in the study area, here we utilize the constructed model to project community composition under two climate change scenarios.

**FIGURE 2:**
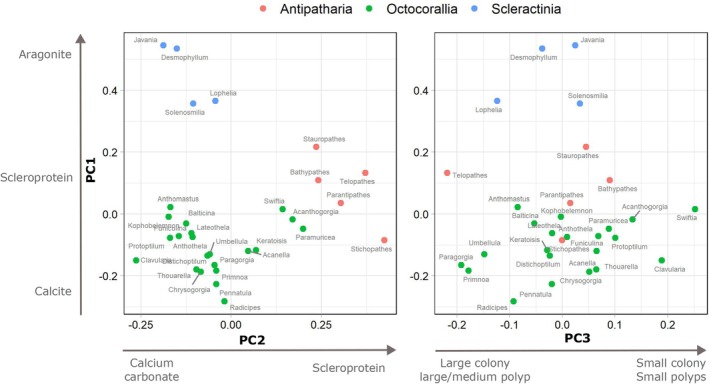
Two‐dimensional representation of the trait space defined by three trait axes (PC1, PC2, PC3) based on the Principal Coordinate Analysis. Points represent positions of the 30 coral genera in the trait space.

Our model domain was restricted to areas with environmental data (under present conditions) analogous to those used to train the HMSC model (Rakka et al. 2025). The model was developed using data on presence and pseudoabsence of 30 deep‐water coral genera, including representatives of three main coral taxonomic groups (Scleractinia, Antipatharia, Octocorallia, Table S1.1). Coral data were collected during 103 ROV and towed camera dives that took place between 2013 and 2019 (Figure S1.1). The collected video footage was then analyzed to determine coral presence and absence in cells of a 1‐km grid that was overlaid on the study area. Corals were identified to genus because identification of organisms to the species level using imagery alone is difficult and often subjective. Through video analysis, we identified a pool of 49 coral genera, 19 of which were excluded as they occurred in less than 2.5% of the sampling locations. Since we did not systematically sample the entire area overlaid by the grid cells using standardized protocols, we consider absences as pseudoabsences (Rakka et al. 2025). The model was developed with six environmental variables and a proxy of sampling effort, all of which were included as fixed effects. The environmental variables consisted of three terrain variables (describing the seafloor) including aspect, broad Topographic Position Index (TPI, 20 km) and mud content, which was used as a proxy of substrate type, as well as three oceanographic variables (describing the water column), including bottom temperature, salinity, and current velocity. Bottom temperature was added as a quadratic effect to allow for nonlinear responses. Oceanographic variables were obtained by averaging data available from hindcasts of the hydrodynamic model FVCOM‐GoM/GB v3 (Chen et al. 2011) over the period 2000–2014. Aspect and TPI were estimated from bathymetry data provided by the global multiresolution topography synthesis (Ryan et al. 2009). For the construction of the HMSC model, we considered all variables associated with climate stressors in deep‐sea environments including bottom temperature, salinity, pH, calcite and aragonite saturation, dissolved oxygen, and particulate organic carbon concentration (Rakka et al. 2025). However, most of these variables were excluded from the final model due to correlations with bottom temperature and salinity (Figure S1.2). More information on the sources of each variable and variable correlation is available in Supporting Information (Table S1.2 and Figure S1.2, respectively). Random effects were included to account for spatial autocorrelation at the levels of grid cell and dive. In addition, the HMSC model contained information on coral traits, including coral skeletal material (fuzzy variable, expressed as an approximate percentage of three categories: aragonite, calcite, scleroprotein), colony height (continuous) and polyp diameter (continuous). Data for these traits were compiled from specimens collected during the surveys and from the literature (Tables S1.3 and S1.4). These traits were first used to construct a multidimensional trait space by performing a Principal Coordinate Analysis (PCoA), which collapsed the information of the five traits into three trait axes: PC1 described a gradient from corals with large colonies, small polyps, and calcite skeletons, to coral genera with small colonies, large polyps, and aragonite skeletons; PC2 represented a gradient from corals with small colonies that use calcium carbonate (calcite or aragonite) to larger corals that use scleroprotein; and PC3 represented a gradient from corals with large colonies and large/medium polyps to those with smaller colonies and polyps (Figure 2). The PCoA was performed using the Gower distance, which allows the inclusion of noncontinuous traits. The trait space was subsequently used to estimate indices of trait diversity as metrics of change. We also used the position of each coral genus on the three principal axes of the PCoA (PC1, PC2, PC3, Figure 2) as input to the HMSC model instead of individual traits. This allowed us to eliminate multicollinearity, thus addressing correlations and trade‐offs among the five coral traits.

The HMSC model was built with Bayesian inference, using the Bernoulli distribution with a probit link function. Models were fitted using Markov chain Monte Carlo techniques, resulting in 1000 posterior samples (4 chains, 250 samples per chain, thinned by 100). Model convergence was satisfactory (Table S1.5), and the model explained 46% of the total variability in coral occurrences. Oceanographic variables explained 69.8% of this variability (bottom temperature: 25.3%, bottom salinity: 37%, bottom current velocity: 4.5%), and terrain variables explained 9.6% (Figure S1.3). Ten‐fold cross‐validation was performed to assess model performance using the index of Area Under the Curve (AUC). This process revealed satisfactory performance (AUC > 0.7) for 29 of the 30 coral genera, and excellent performance (AUC > 0.8) for 26 genera. More information on model fit and the results of cross‐validation are included in the Supporting Information (Figures S1.4–S1.6, respectively).

### 2.3 Predictions of Coral Communities Under Future Scenarios

As the available environmental data from the FVCOM‐GoM model do not include predictions under climate change scenarios, we obtained projections of the three oceanographic variables using the delta method. This method consisted of adding the interpolated mean differences (delta values) between present and future conditions from other models to the available data for present‐day conditions obtained from the FVCOM‐GoM/GB model.

We obtained delta values from two independent data sources: the Bio‐ORACLE database (Assis et al. 2024), and data from a regional ocean model developed for the NW Atlantic (Alexander et al. 2020). Bio‐ORACLE provides environmental layers for current and future conditions on a 0.05° global grid, which corresponds to approximately 4 × 5.5 km grid in our area. Future projections available from Bio‐ORACLE were compiled from delta values between historical (2000–2014) and future (2100) conditions based on the SSP5.8.5 scenario of an ensemble of 11 Earth System Models (ESM) provided by the Sixth Phase of the Coupled Model Intercomparison Project (CMIP6) (Assis et al. 2024). This dataset was chosen as it represents the most recent projections provided by the CMIP6 that reproduce historical physical and biogeochemical properties of the global ocean better than CMIP5 (Hausfather et al. 2022). However, our study takes place in a hydrodynamically complex region where the local circulation is often not well represented in global ESMs, leading to pronounced warming predictions (Laurent et al. 2021). To address this limitation, we used an additional dataset obtained from a regional ocean model that better simulates the local circulation patterns of the study area. This model (Alexander et al. 2020) is a high‐resolution regional ocean model (ROMS) developed for the Northwest Atlantic Ocean, with a 7‐km grid resolution. Future projections in this model represent the RCP8.5 scenario for 2100, and delta values originate from an ensemble of three global climate models provided by CMIP5.

**FIGURE 3:**
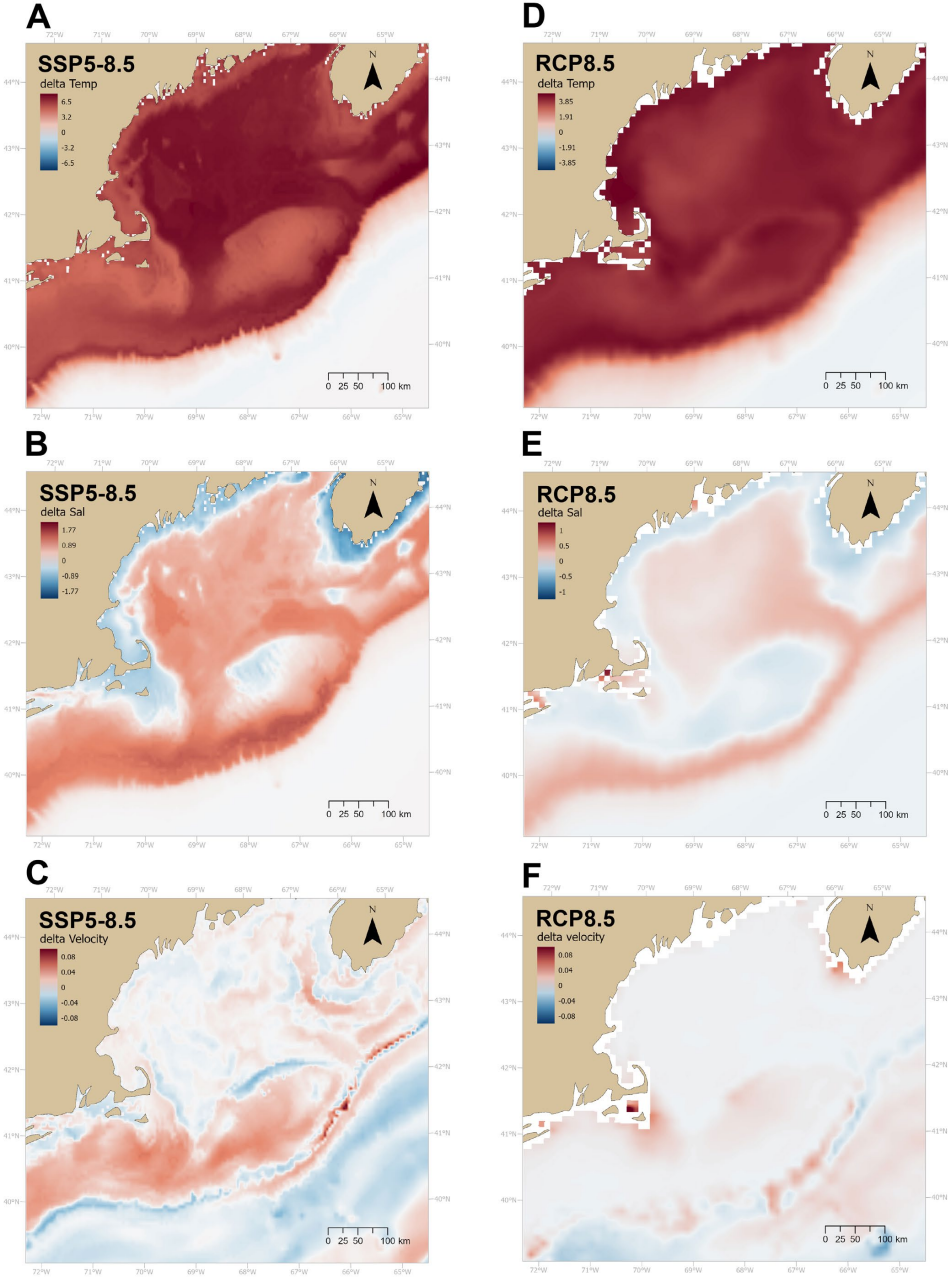
Delta values for Temperature (Temp, °C), Salinity (Sal), Velocity (Current, m/s) under two climate projections: SSP5‐8.5 obtained from Bio‐ORACLE (Assis et al. 2024) and RCP8.5 (Alexander et al. 2020) for 2100. Note the different scales in color for temperature and salinity.

To calculate our projections, we first extracted the delta values for the three oceanographic variables from both data sources (corresponding to SSP5‐8.5 scenario for Bio‐ORACLE and RCP8.5 scenario for the ROMS model). We then downscaled the delta values to the grid of our model using bilinear interpolation and added them to the historical data obtained from the FVCOM‐GoM model. Resulting projections for each environmental variable and climate change scenario are included in the Supporting Information (Figure S2.1), while the delta values are presented on Figure 3. We identified grid cells that are outside of the range of the environmental covariates with which the HMSC model was trained with the R package *dsmextra* (Bouchet et al. 2020) (Table S2.1, Figures S2.2–S2.3). Since predicting in nonanalogous conditions can interfere with model performance, we used this analysis to determine whether nonanalogous areas overlapped with areas of high model uncertainty (see Metrics of ecological change for a definition of uncertainty). We then used the environmental projections to generate predictions (*n* = 1000) of the occurrence (presence/absence) of the 30 coral genera in our study area, under present and future conditions according to each of the three climate scenarios (present, SSP5‐8.5, RCP8.5). These predictions were generated by randomly drawing 1000 samples from the posterior predictive distribution, which directly provides presence/absence data. Using this data, we estimated the probability of occurrence of each coral genus by dividing the sum of the number of occurrences of each genus by the total number of predictions (*n* = 1000) for each grid cell and climate scenario. The model was fitted in R v2.2 (R Core Team 2022) using the *Hmsc* package (Tikhonov et al. 2020).

### 2.4 Metrics of Ecological Change

To compare present and projected coral communities, we examined three metrics of ecological change: changes in composition, complexity (taxonomic diversity) and function (trait diversity). Compositional differences between communities under the present and future scenarios were assessed with the Bray‐Curtis (BC) dissimilarity index. We calculated the index for each cell of the model grid as follows:

$${\mathrm{BC}}_{\mathrm{jkg}}=\frac{\sum_{i}\left|x_{\mathrm{ij}}-x_{\mathrm{ik}}\right|}{\sum_{i}\left|x_{\mathrm{ij}}+x_{\mathrm{ik}}\right|}$$

where $x_{\mathrm{ij}}\;\text{and}\;x_{\mathrm{ik}}$ refer to the probability of occurrence of coral genus *i* in a grid cell *g*, under present (*j*) and future (*k*) conditions, respectively. Since the probability of occurrence is not a measure of abundance, we can only use the Bray‐Curtis index as an index of the overall changes in the predicted probability of occurrence of coral genera within the community. Calculations for this index were made using the R library *vegan* (Oksanen et al. 2025). In addition, we assessed the delta values in the probability of occurrence of individual genera (Figures S3.1 and S4.1).

Changes in taxonomic diversity were assessed by estimating deltas for the total number of coral genera in each cell (Taxonomic richness). Taxonomic richness (Tric) under future scenarios is presented as percent change (delta) related to Tric under present conditions. To explain patterns in delta Tric, we further assessed the range shifts of individual genera by estimating the total area of suitable habitat, as well as the centroid of the range and average depth for each coral genus under each scenario.

Additionally, we calculated three indices of trait diversity: functional richness, functional evenness, and functional dispersion, as well as the community weighted mean (CWM) for each of the coral trait axes mentioned above. Functional richness (Fric) provides a measure of the total volume of the multidimensional trait space occupied by the community (de Bello et al. 2021). To simplify interpretations, this index was standardized to the maximum functional richness of the pool that included all the coral genera included in our study. Functional evenness (Feve) reflects whether coral genera are evenly spread in the trait space or if they accumulate in specific areas. Lastly, functional dispersion (Fdis) represents how distant species of a community are from the CWM (de Bello et al. 2021). All trait diversity indices were calculated using the R package *fundiversity* (Grenié and Gruson 2023).

The calculation of metrics relating to species' range shifts and diversity metrics from predictions of correlative models requires the transformation of the predicted probabilities of occurrence (decimal values ranging from 0 to 1) to binary data (presence‐absence). Sensitivity analysis is usually conducted to define the threshold between presence and absence. However, sensitivity analysis can mask the probabilistic nature of the predictions and their associated uncertainty (Guillera‐Arroita et al. 2015; Santini et al. 2021). The Bayesian approach used herein allowed us to calculate diversity metrics directly from the predicted binary data provided by the posterior predictive distribution of the HMSC model in each scenario. Due to the large computation times required to estimate some of these indices, a subsample of 300 datasets was used to calculate trait diversity metrics and CWM, whereas the total number of predictive samples (*n* = 1000) was used to calculate Tric and range shift metrics. We then calculated the median, as well as the 25th and 75th percentiles for each metric. The median and percentiles were chosen over the mean and standard deviation to provide an indication of skewness. In addition, the percentiles and their difference, known as the interquartile range (IQR), served as indicators of uncertainty.

## 3 Results

### 3.1 Projected Environmental Changes

Substantial warming was predicted in both projections, particularly in the deeper basins within the GOM, as well as on the shelf break and the upper continental shelf (Figure 3). The level of warming in these areas was lower in the RCP8.5 (3°C–4°C) compared to the SSP5‐8.5 scenario (5°C–6°C) and decreased with depth (Figure 3). Similar trends were observed in salinity, with the maximum increase observed over the shelf break and upper continental slope (SSP5‐8.5: 1–1.7, RCP8.5: 0.3–0.4). In RCP8.5, the maximum increase in both temperature and salinity within the GOM was observed in the southeast portion of the Gulf and in the Northeast Channel, while under SSP5‐8.5, the increase in temperature was more uniform. Changes in bottom current velocity were localized and very minor in both projections (Figure 3C,F).

### 3.2 Changes in Probability of Occurrence and Taxonomic Richness

**FIGURE 4:**
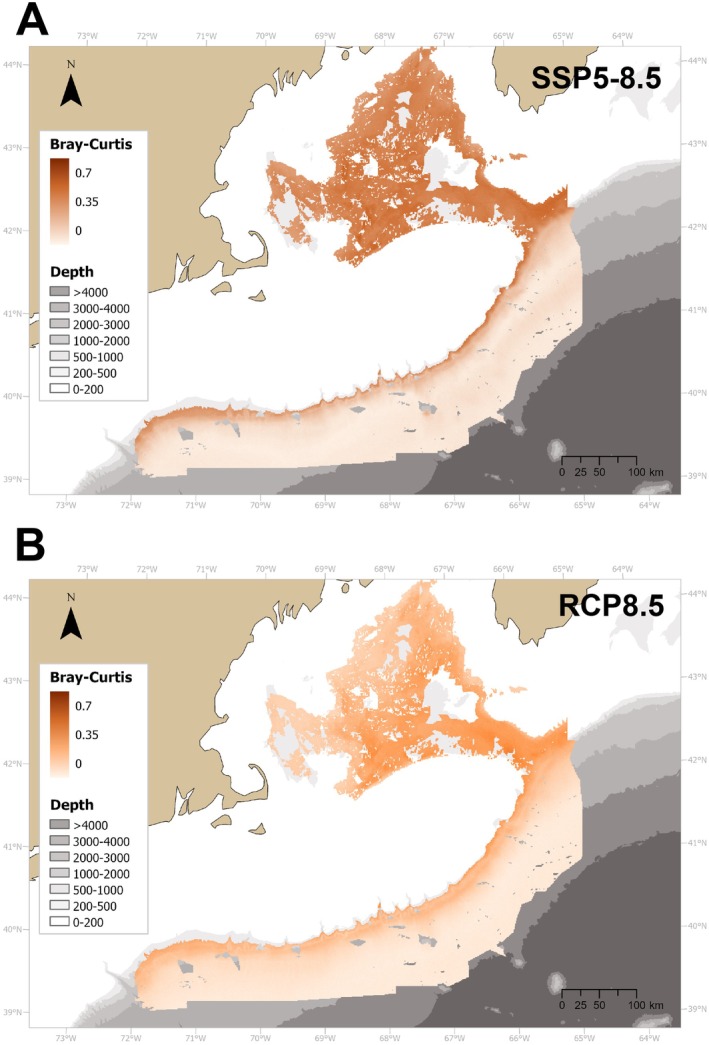
Bray–Curtis dissimilarity index highlighting differences in the probability of occurrence of 30 deep‐water coral genera between present conditions and projections under the SSP5‐8.5 (A), and RCP5.8 scenarios (B) for 2100.

Under SSP5‐8.5, the main changes in the frequency of occurrence of corals occurred in the upper 1000–1500 m, as indicated by the highest BC dissimilarity index (BC = 0.4–0.5) in the GOM and the Northeast Channel, and its slightly lower values (BC = 0.3–0.4) along the upper continental slope (Figure 4). The values of the BC dissimilarity index decreased at greater depths. These changes were associated with a notable decrease in the frequency of occurrence of several coral genera, including *Paragorgia*, *Paramuricea*, *Primnoa*, *Pennatula*, and *Acanella* within the GOM, as well as *Acanthogorgia, Anthomastus, Anthothella, Desmophyllum, Keratoisis, and Paragorgia* on the continental slope (Figures S3.1, S4.1). Overall, the dissimilarity index was lower in the RCP8.5 than in the SSP5‐8.5 scenario, with the former displaying maximum values (BC = 0.3–0.4) in the southeastern GOM, and slightly lower values along the upper slope (BC = 0.2–0.3, Figure 4). The genus‐specific results under the RCP8.5 scenario corroborated those under SSP5‐8.5, except for the genus *Primnoa* (Figures S3.1, S4.1).

**FIGURE 5:**
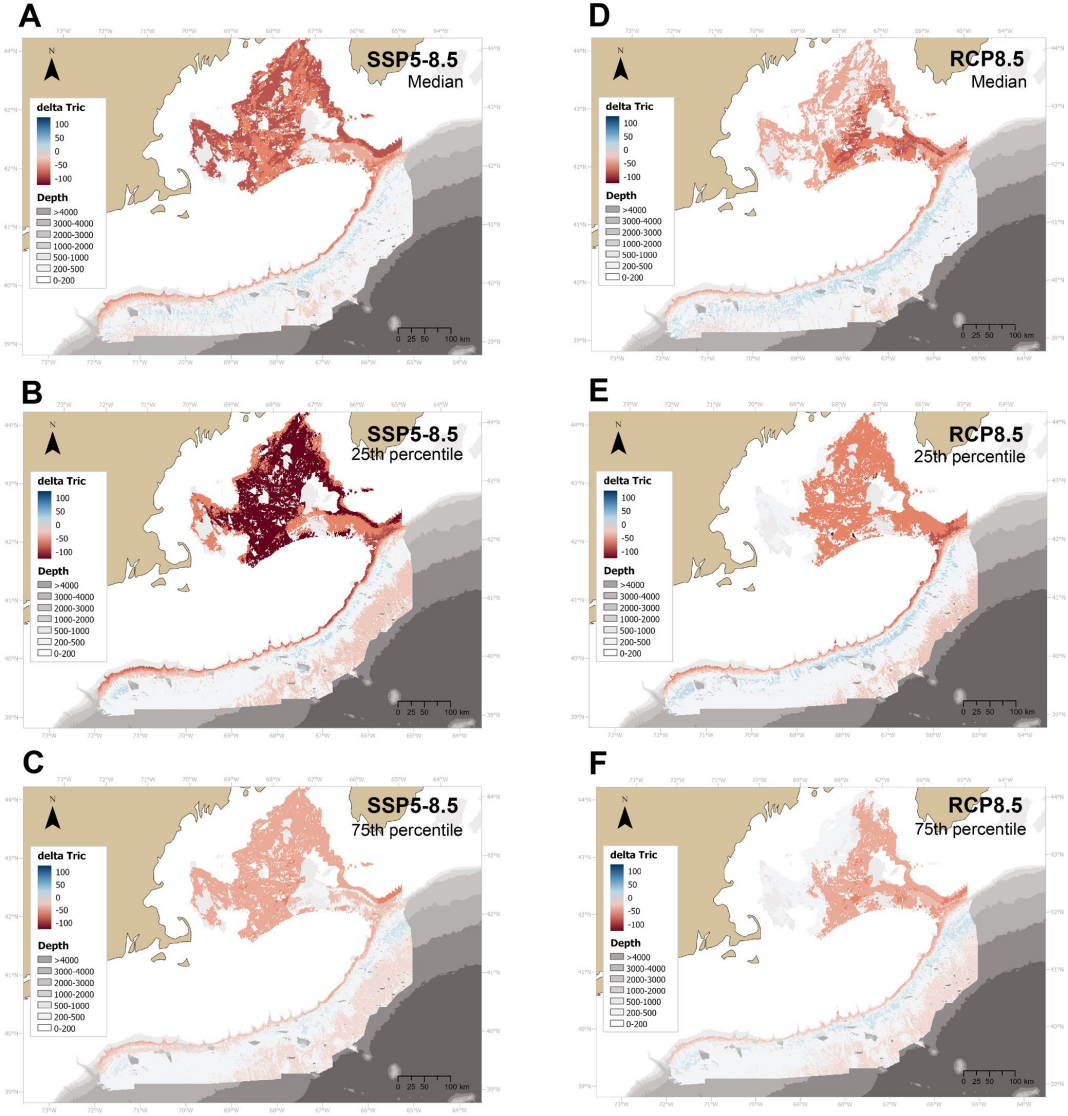
Delta values of taxonomic richness (Tric) throughout the study area under two climate change scenarios (SSP5‐8.5 and RCP8.5) for 2100. Top plots (A, D) represent the median, middle plots (B, E) represent the 75th percentile and bottom plots (C,F) represent the 25th percentile.

Both projections predicted a large decrease (by 25%–60%) in taxonomic richness (Tric) within the GOM and along the shallowest 1000 m of the continental slope. Under the SSP5‐8.5 scenario, this decrease occurred throughout the GOM, while under RCP8.5 the decrease was more localized to Georges Basin and the Northeast Channel (Figure 5). Although the uncertainty regarding the degree of losses under SSP5‐8.5 was high (Figure S5.1), trends were similar for the 25th and 75th percentiles (Figure 5). Under RCP8.5, uncertainty was generally lower, except in the northwest basins of the GOM where changes in Tric varied from minimal (0%–5%) to moderate (10%–20%). Both models predicted gains in Tric (by 10%–15%) between 1000 and 2000 m, corroborating the predicted increase in the frequency of occurrence of several coral genera in these areas. Overall, higher uncertainty was observed in the upper 1000–1500 m (Figures S5.2, S5.3), which coincides spatially with areas with nonanalogous conditions (Figures S2.2, S2.3).

### 3.3 Genera‐Specific Changes

**FIGURE 6:**
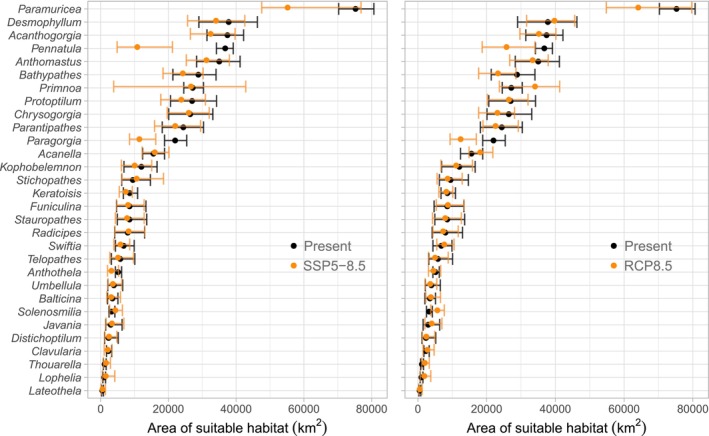
Total area of suitable habitat of 30 deep‐water coral genera throughout the study area under present conditions (black color) and two climate change projections (SSP5‐8.5 and RCP8.5, orange color) for 2100. Points represent the median, while lines represent the interquantile range between the 25th and 75th percentile calculated from 300 predicted datasets for each projection.

**FIGURE 7:**
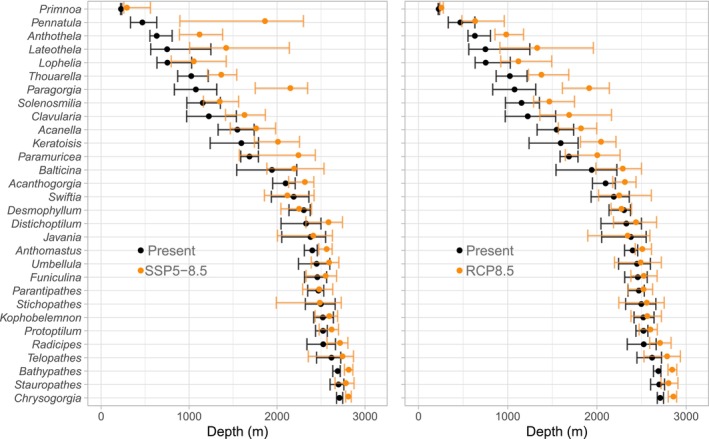
Average depth of suitable habitat of 30 deep‐water coral genera throughout the study area under present conditions (black color) and two climate change projections (SSP5‐8.5 and RCP8.5, orange color) for 2100. Points represent the median, while lines represent the interquantile range between the 25th and 75th percentile calculated from 300 predicted datasets for each projection.

In both SSP5‐8.5 and RCP8.5 scenarios, the total area of suitable habitat decreased only for *Pennatula* and *Paragorgia*, as indicated by a nonoverlap of Interquantile Range (IQR) between present and future projections (Figure 6). Large shifts were observed in the average depth of suitable habitat in both projections, with many genera displaying shifts to greater depths (Figure 7). These shifts were more common in genera whose suitable habitat under present conditions occurred in the upper 1000–1500 m, such as *Pennatula*, *Anthothella*, and *Paragorgia*. However, shifts to deeper areas were also observed in coral genera, such as *Telopathes* and *Chrysogorgia*, where suitable habitat was initially restricted to greater depths (Figure 7). Suitable habitat for some genera displayed bimodal depth distributions; thus, local shifts may be masked if only mean depth is considered. Although the IQR varied among genera and between projections, the shifts to deeper waters persisted in both projections.

Southward shifts of suitable habitat were observed for some genera in both projections, with general agreement between scenarios. These included *Acanthogorgia*, *Keratoisis*, *Acanella, Anthothella, Paragorgia, Pennatula*, *and Primnoa* (Figure S6.1), most of which showed high occurrence at higher latitudes in our study area under present conditions.

### 3.4 Changes in Trait Diversity

**FIGURE 8:**
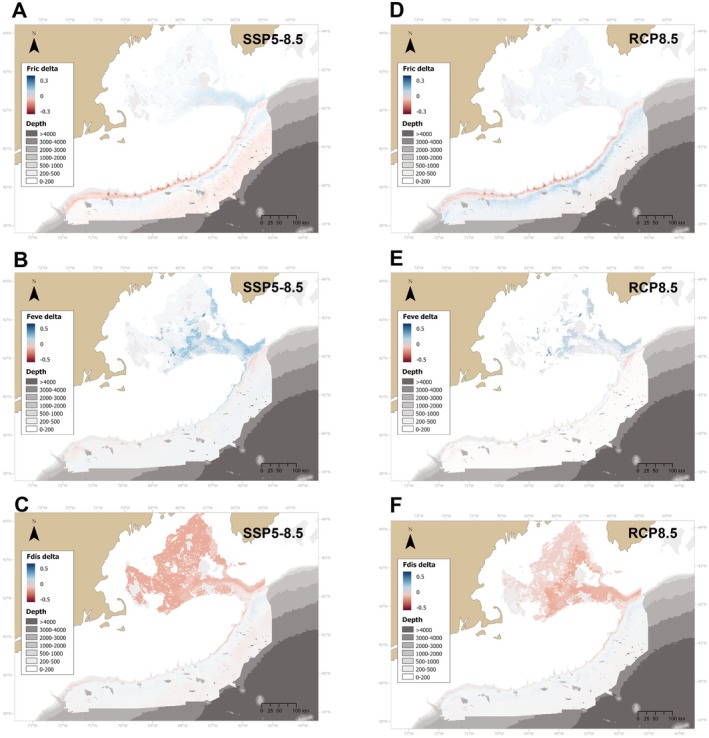
Delta values (future projection values minus present values) of trait diversity indices, including Functional richness (Fric), Functional evenness (Feve) and functional dispersion (Fdis) for deep‐water coral communities in the study area under two climate change projections: SSP5‐8.5 and RCP8.5 for 2100.

Functional richness (Fric) decreased substantially (by 0.05–0.2, corresponding to 15%–60% of the initial Fric) on the upper continental slope under both projections (Figure 8), but remained highest in the southern canyons. A slight increase was projected under SSP5‐8.5 in the NEC and Georges Basin (0.02–0.05), which represents a substantial change considering the overall low functional richness of the area (Fric = 0.01–0.02, Rakka et al. 2025). Increases of Fric were also observed at the lower bathyal depths (Figure 8). The IQR for Fric was highest in the NEC and adjacent canyons under the SSP5‐8.5 scenario but was low throughout the area under RCP8.5 (Figure S5.7A,B). In both projections, the 25th and 75th percentiles confirmed the general trend (Figure S5.4).

Functional evenness (Feve) displayed very small changes under both projections (Figure 8). Under SSP5‐8.5, Feve increased slightly in the southeastern GOM, NEC, and adjacent canyons, while under the RCP8.5 scenario this increase was restricted to the southeastern GOM (Figure 8). Moreover, both projections displayed a small decrease of Feve (0.01–0.05) in the deeper areas of the NEC. Functional dispersion (Fdis) decreased substantially within the GOM for both models; however, uncertainty was high (Figure S5.6). The uncertainty of the three trait diversity indices was higher in the upper 500 m (Figure S5.7–S5.9) and mostly overlapped with areas with non‐analogous conditions, except for Fric in the RCP8.5 scenario in which uncertainty remained constant throughout the domain (Figure S5.9).

**FIGURE 9:**
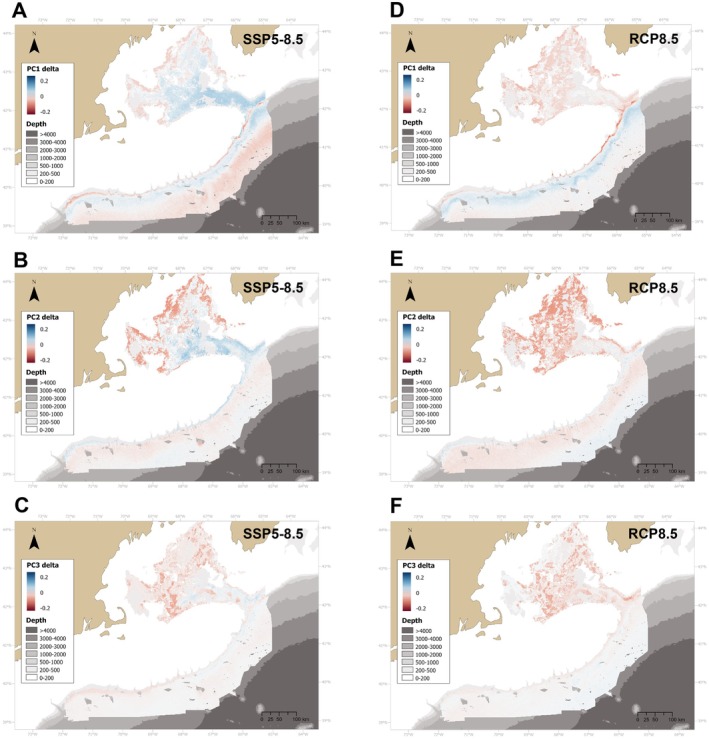
Delta values (future projection values minus present values) of community‐weighted mean of three coral axes for deep‐water coral communities in the study area under two climate change scenarios: SSP5‐8.5 and RCP8.5 for 2100. PC1 represents a gradient from corals with large colonies, small polyps that use primarily calcite to build their skeleton to coral genera with small colonies, large polyps that use aragonite to build their skeleton. PC2 represents a gradient from corals with small colonies that use calcite to larger corals that use scleroprotein. PC3 represents a gradient from corals with large colonies and large polyps to those with smaller colonies and polyps.

Projections of the community weighted mean of the three trait axes were highly variable between scenarios and were associated with very wide IQR values (Figure S5.10–S5.13). In both projections, there was a large decrease in the values of the calcite‐aragonite axis (PC1) on the upper continental slope, and an increase at 1000–2000 m depth (Figure 9). Moreover, PC2 values (calcite‐scleroprotein axis) moderately increased on the upper continental slope, and decreased notably at greater depths in both projections (Figure 9). The results for PC1 and PC2 indicate an increase in corals utilizing calcite on the upper continental slope, and an increase of corals utilizing aragonite and scleroprotein at greater depths. Lastly, PC3 values decreased in specific areas within the GOM, such as the southeastern region of Wilkinson Basin, indicating an increase in corals with larger colonies in this region (Figure 9). However, none of these patterns were maintained under the 25th and 75th percentiles (Figures S5.10–S5.12).

## 4 Discussion

In our study, we projected changes in the composition, species diversity, and trait diversity of communities of deep‐water corals under two climate change scenarios. We identified extensive loss of currently occurring species and a decrease in functional richness in the upper 1000 m of the continental shelf and slope. These changes were mainly due to shifts of suitable habitat to greater depths, leading to diversity gains on the lower continental slope and bathyal zone. Although several oceanographic variables were correlated with coral traits under present conditions in our model, we did not identify any prevailing traits in the projected coral communities, indicating that responses were mostly genus‐specific.

The greatest loss of species occurred within the GOM, and along the upper continental slope (500–1000 m). Under climate projections, taxonomic richness within the GOM diminished to 1–2 genera and functional richness approached zero. Importantly, the upper continental slope, which hosts the highest diversity of coral species in the area (Rakka et al. 2025), lost 20%–30% of coral genera. Although substantial taxonomic richness (4–7 genera) persisted at this depth, functional richness decreased and the suitable habitat for coral genera that create dense coral gardens, such as *Paramuricea, Paragorgia, Primnoa*, and *Thouarella*, shifted to greater depths. These results corroborate previous studies which have identified mesopelagic depths (200–1000 m) as susceptible to biodiversity loss due to escalating shifts in environmental conditions worldwide (Brito‐Morales et al. 2020). Under both climate projections, mesopelagic depths were characterized by extreme warming and an increase in salinity. Even though salinity was positively correlated with the presence of corals that utilize aragonite for their skeleton in our model, we observed minor increases in the occurrence of these genera, thus underscoring temperature as the main driver of the observed patterns in the upper 1000 m. Previous studies have highlighted the rapid and extreme warming which has already impacted species locally (e.g., Pershing et al. 2015), and have raised concerns about the future of ecosystems in this area (Staudinger et al. 2019; Siedlecki et al. 2021; Pershing et al. 2021). Our results highlight that these changes are not restricted to shallow depths, and warming may have substantial ecological effects from the continental shelf to the bathyal zone.

Despite the substantial decrease in taxonomic richness in the shallower depths, the total area of suitable habitat decreased for only two coral genera in our study. This is contrary to previous studies in the region (Morato et al. 2020; Gasbarro et al. 2022) and elsewhere (Anderson et al. 2022), which reported major decreases in suitable habitat for some deep‐water coral species. Projected shifts to deeper areas have been previously reported in this region for the deep‐water corals *Desmophyllum pertusum* (formerly *Lophelia pertusa* ), *Acanthogorgia armata* , and *Acanella arbuscula* (Gasbarro et al. 2022; Morato et al. 2020). However, to our knowledge, our study is the first to report such large bathymetric shifts in the distribution of deep‐sea benthic communities under climate change projections. Shifts to greater depths due to recent warming have been observed for many other benthic invertebrates in coastal ecosystems (e.g., Poloczanska et al. 2013; Hiddink et al. 2015), but in the deep sea such observations have been mostly restricted to fishes (Dulvy et al. 2008; Nye et al. 2009). Nevertheless, similar shifts and the existence of deep‐sea refugia during adverse climatic events have been reported throughout geological time for several benthic species (Schneider 2018), including corals (Stanley et al. 2018; Campoy et al. 2023). Our finding parallels historical patterns of species persistence under unfavorable changes and highlights the importance of greater depths as critical refugia for biodiversity in the face of climate change. Surprisingly, we did not observe substantial shifts to higher latitudes. This is likely due to our limited model domain. Wang et al. (2022) have reported an increase of suitable habitat for the deep‐water coral *Paragorgia arborea* along the Scotian Shelf. Thus, it is possible that areas north of our domain may include additional suitable habitat for some of our target coral genera that we did not capture.

Bathymetric shifts in taxonomic and trait diversity will inevitably lead to changes in ecosystem functions and services. Benthic communities at mesopelagic depths often support high diversity of invertebrates and fishes (De Clippele et al. 2015; Buhl‐Mortensen et al. 2017; Gallego et al. 2024). The loss of large habitat‐forming species in these communities will have negative impacts on their associated fauna. This is particularly important for associates with limited dispersal capacity, and for obligate habitat specialists, which rely exclusively on certain species or habitats to survive. Further cascading impacts on local productivity, biogeochemical cycles, and food webs may occur. In addition, the increase of species diversity at greater depths can alter historical species interactions and impact community dynamics and ecosystem function (Pinsky et al. 2020). It may also intensify competition, leading to novel community dynamics and evolutionary adaptations. Although species interactions are well studied in coastal and shallow‐water ecosystems (e.g., Johnson et al. 2011; Kortsch et al. 2012), very little is known about these relationships in the deep sea, where knowledge is limited to organismal‐ and species‐level processes (Levin 2021). As our findings highlight, our capacity to predict the potential impacts of climate change in these remote habitats is extremely limited, and more studies are essential to better understand potential future impacts.

Despite the projection of suitable habitat in greater depths, such range shifts may not be realistic for all coral genera. Since corals are calcifiers, their bathymetric distribution is very often limited by the aragonite/calcite saturation horizon (Lunden et al. 2013). In our study, under present conditions, areas deeper than 1000 m were bathed in cool, fresh subpolar water masses. In both climate projections, these water masses were replaced by warmer, saltier waters, which are more favorable for coral calcification, particularly for corals that use aragonite. Previous studies have shown that under future projections the aragonite saturation horizon in this region remains close to 1000 m (Zheng and Cao 2014), while the calcite saturation horizon extends even deeper (Wei et al. 2020). These projections corroborate the possibility of a shift of suitable habitat to greater depths for some corals in our study area. However, as depth increases, food availability often decreases, and it is unknown whether food supply at these depths can sustain deep‐water corals metabolically. This is particularly relevant in the context of climate change, since according to climate projections food availability will decrease in deep‐sea environments (Levin and Le Bris 2015; Sweetman et al. 2017). Importantly, it is unknown whether the local hydrodynamics and biological traits of the corals will allow them to disperse to deeper areas with suitable habitats. In the absence of connective pathways, such shifts will not be possible and affected coral genera may become locally extinct. In addition, although we incorporated available information on substrate type in our model, this variable does not adequately represent the availability of hard substrate. Therefore, even under favorable conditions for larval dispersal, the available substrate for settlement in deeper areas may be limiting. Lastly, in our model we assumed that corals have a stable niche; however, some coral genera may be able to adapt to changing environmental conditions. Unfortunately, our knowledge of their physiological tolerance is extremely limited. Therefore, more studies are essential to better understand the physiological and biological characteristics of these organisms, as well as their potential to disperse under future conditions.

Although our results provide novel insights into climate‐induced changes in deep‐sea communities, they must be interpreted with caution. The projections we developed are limited by the regional biological data used in training the HMSC model, which might not cover the entire niche of our target coral genera, possibly explaining differences with previous studies with larger spatial extents (Morato et al. 2020). In addition, both climate projections included a large quantity of non‐analogous environmental conditions, mostly located in the shallowest 1000 m, which led to high model uncertainty for these areas and depths. This is a general limitation in this region and particularly in the GOM, as climate projections forecast extreme conditions that differ greatly from the current environmental regime (Lotze et al. 2022). This region is characterized by complex circulation patterns, particularly affected by the position of the Gulf Stream, which is not resolved efficiently in many of the available climate projections (Laurent et al. 2021). In our model, we addressed this issue by incorporating environmental projections from different sources, including an oceanographic model with a higher efficiency in simulating the complex regional hydrodynamics (Alexander et al. 2020). We show that despite disagreements between the predictions based on the two projections and high uncertainty, the main patterns persist. Importantly, due to our restricted model domain, our study does not consider expansions of species' distributions from and towards sites outside our study area. Expanding the study area can potentially highlight higher species turnover and more instances of northward expansion of species distributions that could compensate for the loss of coral genera locally. However, such analysis is currently limited by data availability. Despite these important limitations, our study highlights the loss of suitable habitat for several corals of the regional species pool in their current depth range and the decrease of local taxonomic and functional diversity, indicating substantial shifts in ecosystem functions and services.

Our results not only contribute to the limited knowledge of potential impacts on deep‐sea benthic communities but are also particularly useful for conservation. Our study area is in a region where warm and cold oceanographic systems converge, making it extremely relevant for climate‐induced distributional shifts. Therefore, monitoring the status of community composition of deep‐sea species, and particularly habitat‐forming taxa such as corals, in this region is imperative. In addition, climate‐induced changes in local circulation patterns, and potential shifts of the position of the Gulf Stream, might alter present connectivity pathways and facilitate or prevent range shifts (van Gennip et al. 2017). While many studies emphasize the importance of considering such complex ecological processes in deep‐sea research and conservation (Le et al. 2017; Levin et al. 2020; Howell et al. 2021), efforts so far have largely focused on single species and habitats. Moreover, in the deep sea, it is common practice to target newly discovered, rich benthic communities for protection. However, our results highlight the importance of incorporating climate considerations into conservation planning and management. Designated protected areas in mesopelagic depths may face substantial challenges in the future, and ecosystem functions and services that are currently associated with these depths might be lost or transition deeper. Potential climatic refugia may exist at greater depths, but we show that these depths are subject to warming and other climate change stressors. Despite the high costs associated with studying the deep ocean, there is an increasing amount of data emerging, and several efforts aim to enhance the capacity for observing the deep ocean globally (Levin et al. 2019; Howell et al. 2021; Bell et al. 2022). Directing those efforts to study impacts at higher ecological scales is essential to better understand and address the broader ecological shifts occurring in this realm.

## Author Contributions

**M. Rakka:** formal analysis, investigation, writing – original draft. **A. Metaxas:** funding acquisition, investigation, supervision, writing – review and editing. **M. Nizinski:** funding acquisition, investigation, writing – review and editing.

## Conflicts of Interest

The authors declare no conflicts of interest.

## Supporting information


**Data S1:** gcb70407‐sup‐0001‐Supinfo1.pdf.

**Data S2:** gcb70407‐sup‐0002‐Supinfo2.pdf.

**Data S3:** gcb70407‐sup‐0003‐Supinfo3.pdf.

**Data S4:** gcb70407‐sup‐0004‐Supinfo4.pdf.

**Data S5:** gcb70407‐sup‐0005‐Supinfo5.pdf.

**Data S6:** gcb70407‐sup‐0006‐Supinfo6.pdf.

## Data Availability

The data and code that support the findings of this study are openly available in Zenodo at https://doi.org/10.5281/zenodo.16109708. The bathymetry data used to construct terrain variables were obtained from the Global Multi‐Resolution Topography Data Synthesis at https://www.gmrt.org/ (v 4.0). Oceanographic data were obtained from the University of Massachusetts—Dartmouth at http://www.smast.umassd.edu:8080/thredds/catalog/models/fvcom/NECOFS/Archive/Seaplan_33_Hindcast_v1/catalog.html). Environmental projections under different climate scenarios were obtained from BioORACLE at https://bio‐oracle.org (v 3).

## References

[gcb70407-bib-0001] Alexander, M. A. , S. Shin , J. D. Scott , E. Curchitser , and C. Stock . 2020. “The Response of the Northwest Atlantic Ocean to Climate Change.” Journal of Climate 33, no. 2: 405–428. 10.1175/JCLI-D-19-0117.1.

[gcb70407-bib-0002] Anderson, O. F. , F. Stephenson , E. Behrens , and A. A. Rowden . 2022. “Predicting the Effects of Climate Change on Deep‐Water Coral Distribution Around New Zealand—Will There Be Suitable Refuges for Protection at the End of the 21st Century?” Global Change Biology 28, no. 22: 6556–6576. 10.1111/gcb.16389. 36045501 PMC9804896

[gcb70407-bib-0003] Assis, J. , S. J. Fernández Bejarano , V. W. Salazar , et al. 2024. “Bio‐ORACLE v3.0. Pushing Marine Data Layers to the CMIP6 Earth System Models of Climate Change Research.” Global Ecology and Biogeography 33, no. 4: e13813. 10.1111/geb.13813.

[gcb70407-bib-0004] Auster, P. J. , M. Kilgour , D. Packer , R. Waller , S. Auscavitch , and L. Watling . 2013. “Octocoral Gardens in the Gulf of Maine (NW Atlantic).” Biodiversity 14, no. 4: 193–194. 10.1080/14888386.2013.850446.

[gcb70407-bib-0005] Balch, W. M. , D. T. Drapeau , B. C. Bowler , et al. 2022. “Changing Hydrographic, Biogeochemical, and Acidification Properties in the Gulf of Maine as Measured by the Gulf of Maine North Atlantic Time Series, GNATS, Between 1998 and 2018.” Journal of Geophysical Research: Biogeosciences 127, no. 6: e2022JG006790. 10.1029/2022JG006790. PMC9287075 35865236

[gcb70407-bib-0006] Beazley, L. , E. Kenchington , F. J. Murillo , et al. 2021. “Climate Change Winner in the Deep Sea? Predicting the Impacts of Climate Change on the Distribution of the Glass Sponge Vazella Pourtalesii.” Marine Ecology Progress Series 657: 1–23. 10.3354/meps13566.

[gcb70407-bib-0007] Bell, K. , C. Quinzin , S. Poulton , A. Hope , and D. J. Amon . 2022. 2022 Global Deep‐Sea Capacity Assessment. Ocean Discovery League. 10.21428/cbd17b20.48af7fcb.

[gcb70407-bib-0008] Bindoff, N. , W. W. L. Cheung , J. G. Arístegui , et al. 2019. “Changing Ocean, Marine Ecosystems, and Dependent Communities.” In IPCC Special Report Oceans and Cryospheres in Changing Climate, edited by D. Pörtner , V. Roberts , P. Masson‐Delmotte , et al. IPCC.

[gcb70407-bib-0009] Bouchet, P. J. , D. L. Miller , J. J. Roberts , L. Mannocci , C. M. Harris , and L. Thomas . 2020. “Dsmextra: Extrapolation Assessment Tools for Density Surface Models.” Methods in Ecology and Evolution 11, no. 11: 1464–1469. 10.1111/2041-210X.13469.

[gcb70407-bib-0010] Brickman, D. , M. A. Alexander , A. Pershing , J. D. Scott , and Z. Wang . 2021. “Projections of Physical Conditions in the Gulf of Maine in 2050.” Elementa: Science of the Anthropocene 9, no. 1: e00055. 10.1525/elementa.2020.20.00055.

[gcb70407-bib-0011] Brito‐Morales, I. , D. S. Schoeman , J. G. Molinos , et al. 2020. “Climate Velocity Reveals Increasing Exposure of Deep‐Ocean Biodiversity to Future Warming.” Nature Climate Change 10, no. 6: 576–581. 10.1038/s41558-020-0773-5.

[gcb70407-bib-0012] Buhl‐Mortensen, L. , and P. Buhl‐Mortensen . 2018. “Cold Temperate Coral Habitats.” In Corals in a Changing World. Intech. 10.5772/intechopen.71446.

[gcb70407-bib-0013] Buhl‐Mortensen, P. , L. Buhl‐Mortensen , and A. Purser . 2017. “Trophic Ecology and Habitat Provision in Cold‐Water Coral Ecosystems, Marine Animal Forests.” In The Ecology of Benthic Biodiversity Hotspots, 919–944. Springer International Publishing. 10.1007/978-3-319-21012-4_20.

[gcb70407-bib-0014] Campoy, A. N. , M. M. Rivadeneira , C. E. Hernández , A. Meade , and C. Venditti . 2023. “Deep‐Sea Origin and Depth Colonization Associated With Phenotypic Innovations in Scleractinian Corals.” Nature Communications 14, no. 1: 7458. 10.1038/s41467-023-43287-y. PMC10656505 37978188

[gcb70407-bib-0015] Cartes, J. E. , F. Maynou , E. Fanelli , C. López‐Pérez , and V. Papiol . 2015. “Changes in Deep‐Sea Fish and Crustacean Communities at 1000–2200 m in the Western Mediterranean After 25 Years: Relation to Hydro‐Climatic Conditions.” Journal of Marine Systems 143: 138–153. 10.1016/j.jmarsys.2014.10.015.

[gcb70407-bib-0016] Chen, C. , H. Huang , R. C. Beardsley , et al. 2011. “Tidal Dynamics in the Gulf of Maine and New England Shelf: An Application of FVCOM.” Journal of Geophysical Research: Oceans 116, no. C12: C12010. 10.1029/2011JC007054.

[gcb70407-bib-0017] D'Amen, M. , C. Rahbek , N. E. Zimmermann , and A. Guisan . 2017. “Spatial Predictions at the Community Level: From Current Approaches to Future Frameworks.” Biological Reviews 92, no. 1: 169–187. 10.1111/brv.12222. 26426308

[gcb70407-bib-0018] de Bello, F. , C. P. Carmona , A. T. C. Dias , L. Götzenberger , M. Moretti , and M. P. Berg . 2021. Handbook of Trait‐Based Ecology: From Theory to R Tools. Cambridge University Press. 10.1017/9781108628426.

[gcb70407-bib-0019] De Clippele, L. H. , P. Buhl‐Mortensen , and L. Buhl‐Mortensen . 2015. “Fauna Associated With Cold Water Gorgonians and Sea Pens.” Continental Shelf Research 105: 67–78. 10.1016/J.CSR.2015.06.007.

[gcb70407-bib-0020] Doi, H. , M. Yasuhara , and M. Ushio . 2021. “Causal Analysis of the Temperature Impact on Deep‐Sea Biodiversity.” Biology Letters 17, no. 7: 20200666. 10.1098/rsbl.2020.0666. 34283931 PMC8292016

[gcb70407-bib-0021] Du, J. , W. G. Zhang , and Y. Li . 2021. “Variability of Deep Water in Jordan Basin of the Gulf of Maine: Influence of Gulf Stream Warm Core Rings and the Nova Scotia Current.” Journal of Geophysical Research: Oceans 126, no. 5: e2020JC017136. 10.1029/2020JC017136.

[gcb70407-bib-0022] Dulvy, N. K. , S. I. Rogers , S. Jennings , V. Stelzenmüller , S. R. Dye , and H. R. Skjoldal . 2008. “Climate Change and Deepening of the North Sea Fish Assemblage: A Biotic Indicator of Warming Seas.” Journal of Applied Ecology 45, no. 4: 1029–1039. 10.1111/j.1365-2664.2008.01488.x.

[gcb70407-bib-0023] Emblemsvåg, M. , I. Núñez‐Riboni , H. T. Christensen , A. Nogueira , A. Gundersen , and R. Primicerio . 2020. “Increasing Temperatures, Diversity Loss and Reorganization of Deep‐Sea Fish Communities East of Greenland.” Marine Ecology Progress Series 654: 127–141. 10.3354/meps13495.

[gcb70407-bib-0024] FAO . 2009. International Guidelines for the Management of Deep‐Sea Fisheries in the High Seas, 92. FAO.

[gcb70407-bib-0025] Ferrari, R. , M. Bryson , T. Bridge , et al. 2016. “Quantifying the Response of Structural Complexity and Community Composition to Environmental Change in Marine Communities.” Global Change Biology 22, no. 5: 1965–1975. 10.1111/gcb.13197. 26679689

[gcb70407-bib-0026] Gallego, R. , M. B. Arias , A. Corral‐Lou , et al. 2024. “North Atlantic Deep‐Sea Benthic Biodiversity Unveiled Through Sponge Natural Sampler DNA.” Communications Biology 7, no. 1: 1–14. 10.1038/s42003-024-06695-4. 39160260 PMC11333605

[gcb70407-bib-0027] Gasbarro, R. , D. Sowers , A. Margolin , and E. E. Cordes . 2022. “Distribution and Predicted Climatic Refugia for a Reef‐Building Cold‐Water Coral on the Southeast US Margin.” Global Change Biology 28, no. 23: 7108–7125. 10.1111/gcb.16415. 36054745

[gcb70407-bib-0028] Georges, V. , S. Vaz , P. Carbonara , et al. 2024. “Mapping the Habitat Refugia of *Isidella elongata* Under Climate Change and Trawling Impacts to Preserve Vulnerable Marine Ecosystems in the Mediterranean.” Scientific Reports 14, no. 1: 6246. 10.1038/s41598-024-56338-1. 38485718 PMC10940633

[gcb70407-bib-0029] Gonçalves Neto, A. , J. A. Langan , and J. B. Palter . 2021. “Changes in the Gulf Stream Preceded Rapid Warming of the Northwest Atlantic Shelf.” Communications Earth & Environment 2, no. 1: 74. 10.1038/s43247-021-00143-5.

[gcb70407-bib-0030] Graham, M. H. , B. P. Kinlan , L. D. Druehl , L. E. Garske , and S. Banks . 2007. “Deep‐Water Kelp Refugia as Potential Hotspots of Tropical Marine Diversity and Productivity.” Proceedings of the National Academy of Sciences 104, no. 42: 16576–16580. 10.1073/pnas.0704778104. PMC2034254 17913882

[gcb70407-bib-0031] Gravel, D. , C. Albouy , and W. Thuiller . 2016. “The Meaning of Functional Trait Composition of Food Webs for Ecosystem Functioning.” Philosophical Transactions of the Royal Society, B: Biological Sciences 371, no. 1694: 20150268. 10.1098/rstb.2015.0268. PMC4843690 27114571

[gcb70407-bib-0032] Grenié, M. , and H. Gruson . 2023. “Fundiversity: A Modular R Package to Compute Functional Diversity Indices.” Ecography 2023, no. 3: e06585. 10.1111/ecog.06585.

[gcb70407-bib-0033] Guillera‐Arroita, G. , J. J. Lahoz‐Monfort , J. Elith , et al. 2015. “Is My Species Distribution Model Fit for Purpose? Matching Data and Models to Applications.” Global Ecology and Biogeography 24, no. 3: 276–292. 10.1111/geb.12268.

[gcb70407-bib-0034] Hanz, U. , P. Riekenberg , A. de Kluijver , et al. 2022. “The Important Role of Sponges in Carbon and Nitrogen Cycling in a Deep‐Sea Biological Hotspot.” Functional Ecology 36, no. 9: 2188–2199. 10.1111/1365-2435.14117.

[gcb70407-bib-0035] Hausfather, Z. , K. Marvel , G. A. Schmidt , J. W. Nielsen‐Gammon , and M. Zelinka . 2022. “Climate Simulations: Recognize the ‘Hot Model’ Problem.” Nature 605, no. 7908: 26–29. 10.1038/d41586-022-01192-2. 35508771

[gcb70407-bib-0036] Hennige, S. J. , U. Wolfram , L. Wickes , et al. 2020. “Crumbling Reefs and Cold‐Water Coral Habitat Loss in a Future Ocean: Evidence of “Coralporosis” as an Indicator of Habitat Integrity.” Frontiers in Marine Science 7: 668. 10.3389/FMARS.2020.00668.

[gcb70407-bib-0037] Henry, L.‐A. , and J. M. Roberts . 2017. “Global Biodiversity in Cold‐Water Coral Reef Ecosystems.” In Marine Animal Forests, 235–256. Springer International Publishing. 10.1007/978-3-319-21012-4_6.

[gcb70407-bib-0038] Hiddink, J. G. , M. T. Burrows , and J. García Molinos . 2015. “Temperature Tracking by North Sea Benthic Invertebrates in Response to Climate Change.” Global Change Biology 21, no. 1: 117–129. 10.1111/gcb.12726. 25179407

[gcb70407-bib-0039] Howell, K. L. , A. Hilário , A. L. Allcock , et al. 2021. “A Decade to Study Deep‐Sea Life.” Nature Ecology & Evolution 5, no. 3: 265–267. 10.1038/s41559-020-01352-5. 33239819

[gcb70407-bib-0040] IPCC . 2021. “Climate Change 2021: The Physical Science Basis.” In Contribution of Working Group I to the Sixth Assessment Report of the Intergovernmental Panel on Climate Change. Cambridge University Press. 10.1017/9781009157896.

[gcb70407-bib-0041] Johnson, C. R. , S. C. Banks , N. S. Barrett , et al. 2011. “Climate Change Cascades: Shifts in Oceanography, Species' Ranges and Subtidal Marine Community Dynamics in Eastern Tasmania.” Journal of Experimental Marine Biology and Ecology 400, no. 1: 17–32. 10.1016/j.jembe.2011.02.032.

[gcb70407-bib-0042] Kelly, N. E. , E. K. Shea , A. Metaxas , R. L. Haedrich , and P. J. Auster . 2010. “Biodiversity of the Deep‐Sea Continental Margin Bordering the Gulf of Maine (NW Atlantic): Relationships Among Sub‐Regions and to Shelf Systems.” PLoS One 5, no. 11: e13832. 10.1371/journal.pone.0013832. 21124960 PMC2988790

[gcb70407-bib-0043] Kortsch, S. , R. Primicerio , F. Beuchel , et al. 2012. “Climate‐Driven Regime Shifts in Arctic Marine Benthos.” Proceedings of the National Academy of Sciences 109, no. 35: 14052–14057. 10.1073/pnas.1207509109. PMC3435174 22891319

[gcb70407-bib-0044] Kortsch, S. , R. Primicerio , M. Fossheim , A. V. Dolgov , and M. Aschan . 2015. “Climate Change Alters the Structure of Arctic Marine Food Webs due to Poleward Shifts of Boreal Generalists.” Proceedings of the Royal Society B: Biological Sciences 282, no. 1814: 20151546. 10.1098/rspb.2015.1546. PMC4571709 26336179

[gcb70407-bib-0045] Kwiatkowski, L. , O. Torres , L. Bopp , et al. 2020. “Twenty‐First Century Ocean Warming, Acidification, Deoxygenation, and Upper‐Ocean Nutrient and Primary Production Decline From CMIP6 Model Projections.” Biogeosciences 17, no. 13: 3439–3470. 10.5194/bg-17-3439-2020.

[gcb70407-bib-0046] Lauer, D. A. , and M. L. Reaka . 2022. “Depth Distributions of Benthic and Pelagic Species Highlight the Potential of Mesophotic and Deep Habitats to Serve as Marine Refugia.” Marine Ecology Progress Series 700: 39–52. 10.3354/meps14180.

[gcb70407-bib-0047] Laurent, A. , K. Fennel , and A. Kuhn . 2021. “An Observation‐Based Evaluation and Ranking of Historical Earth System Model Simulations in the Northwest North Atlantic Ocean.” Biogeosciences 18, no. 5: 1803–1822. 10.5194/bg-18-1803-2021.

[gcb70407-bib-0048] Le, J. T. , L. A. Levin , and R. T. Carson . 2017. “Incorporating Ecosystem Services Into Environmental Management of Deep‐Seabed Mining.” Deep Sea Research Part II: Topical Studies in Oceanography 137: 486–503. 10.1016/j.dsr2.2016.08.007.

[gcb70407-bib-0049] Levin, L. A. 2021. “IPCC and the Deep Sea: A Case for Deeper Knowledge.” Frontiers in Climate 3: 720755. 10.3389/fclim.2021.720755.

[gcb70407-bib-0050] Levin, L. A. , B. J. Bett , A. R. Gates , et al. 2019. “Global Observing Needs in the Deep Ocean.” Frontiers in Marine Science 6: 241. 10.3389/fmars.2019.00241.

[gcb70407-bib-0051] Levin, L. A. , and N. Le Bris . 2015. “The Deep Ocean Under Climate Change.” Science 350, no. 6262: 766–768. 10.1126/science.aad0126. 26564845

[gcb70407-bib-0052] Levin, L. A. , C.‐L. Wei , D. C. Dunn , et al. 2020. “Climate Change Considerations Are Fundamental to Management of Deep‐Sea Resource Extraction.” Global Change Biology 26, no. 9: 4664–4678. 10.1111/gcb.15223. 32531093 PMC7496832

[gcb70407-bib-0053] Lin, L. , Y. Liu , Y. Yan , and B. Kang . 2024. “Optimizing Efficiency and Resilience of No‐Take Marine Protected Areas for Fish Conservation Under Climate Change Along the Coastlines of China Seas.” Conservation Biology 38, no. 2: e14174. 10.1111/cobi.14174. 37650435

[gcb70407-bib-0054] Lotze, H. K. , S. Mellon , J. Coyne , et al. 2022. “Long‐Term Ocean and Resource Dynamics in a Hotspot of Climate Change.” Facets 7: 1142–1184. 10.1139/facets-2021-0197.

[gcb70407-bib-0055] Lunden, J. J. , S. E. Georgian , and E. E. Cordes . 2013. “Aragonite Saturation States at Cold‐Water Coral Reefs Structured by *Lophelia Pertusa* in the Northern Gulf of Mexico.” Limnology and Oceanography 58, no. 1: 354–362. 10.4319/lo.2013.58.1.0354.

[gcb70407-bib-0056] Mace, G. M. , K. Norris , and A. H. Fitter . 2012. “Biodiversity and Ecosystem Services: A Multilayered Relationship.” Trends in Ecology & Evolution 27, no. 1: 19–26. 10.1016/j.tree.2011.08.006. 21943703

[gcb70407-bib-0057] Maguire, K. C. , D. Nieto‐Lugilde , M. C. Fitzpatrick , J. W. Williams , and J. L. Blois . 2015. “Modeling Species and Community Responses to Past, Present, and Future Episodes of Climatic and Ecological Change.” Annual Review of Ecology, Evolution, and Systematics 46, no. 1: 343–368. 10.1146/annurev-ecolsys-112414-054441.

[gcb70407-bib-0058] McGinty, N. , A. D. Barton , N. R. Record , et al. 2021. “Anthropogenic Climate Change Impacts on Copepod Trait Biogeography.” Global Change Biology 27, no. 7: 1431–1442. 10.1111/gcb.15499. 33347685

[gcb70407-bib-0059] Montanyès, M. , B. Weigel , and M. Lindegren . 2023. “Community Assembly Processes and Drivers Shaping Marine Fish Community Structure in the North Sea.” Ecography 2023, no. 10: e06642. 10.1111/ecog.06642.

[gcb70407-bib-0060] Morato, T. , J. González‐Irusta , C. Dominguez‐Carrió , et al. 2020. “Climate‐Induced Changes in the Suitable Habitat of Cold‐Water Corals and Commercially Important Deep‐Sea Fishes in the North Atlantic.” Global Change Biology 26, no. 4: 2181–2202. 10.1111/gcb.14996. 32077217 PMC7154791

[gcb70407-bib-0061] Mortensen, P. B. , and L. Buhl‐Mortensen . 2005. “Morphology and Growth of the Deep‐Water Gorgonians Primnoa Resedaeformis and *Paragorgia arborea* .” Marine Biology 147, no. 3: 775–788. 10.1007/s00227-005-1604-y.

[gcb70407-bib-0062] Murillo, F. J. , B. Weigel , M. Bouchard Marmen , and E. Kenchington . 2020. “Marine Epibenthic Functional Diversity on Flemish Cap (North‐West Atlantic)—Identifying Trait Responses to the Environment and Mapping Ecosystem Functions.” Diversity and Distributions 26, no. 4: 460–478. 10.1111/ddi.13026.

[gcb70407-bib-0063] New, A. L. , D. A. Smeed , A. Czaja , et al. 2021. “Labrador Slope Water Connects the Subarctic With the Gulf Stream.” Environmental Research Letters 16, no. 8: e084019. 10.1088/1748-9326/ac1293.

[gcb70407-bib-0064] Nye, J. A. , J. S. Link , J. A. Hare , and W. J. Overholtz . 2009. “Changing Spatial Distribution of Fish Stocks in Relation to Climate and Population Size on the Northeast United States Continental Shelf.” Marine Ecology Progress Series 393: 111–129. 10.3354/meps08220.

[gcb70407-bib-0065] Oksanen, J. , J. Simpson , F. Blanchet , et al. 2025. “Vegan: Community Ecology Package. R Package (Version 2.7–0) [Computer Software].” https://vegandevs.github.io/vegan/.

[gcb70407-bib-0066] Oliver, T. H. , M. S. Heard , N. J. B. Isaac , et al. 2015. “Biodiversity and Resilience of Ecosystem Functions.” Trends in Ecology & Evolution 30, no. 11: 673–684. 10.1016/j.tree.2015.08.009. 26437633

[gcb70407-bib-0067] OSPAR . 2010. Background Document for Coral Gardens, Biodiversity Series, Publication Number: 15486/2010, 39. OSPAR.

[gcb70407-bib-0068] Ovaskainen, O. , and N. Abrego . 2020. Joint Species Distribution Modelling|Ecology and Conservation. Cambridge University Press. https://www.cambridge.org/ca/academic/subjects/life‐sciences/ecology‐and‐conservation/joint‐species‐distribution‐modelling‐applications‐r. 10.1111/2041-210X.13345 PMC7074067 32194928

[gcb70407-bib-0069] Ovaskainen, O. , G. Tikhonov , A. Norberg , et al. 2017. “How to Make More out of Community Data? A Conceptual Framework and Its Implementation as Models and Software.” Ecology Letters 20, no. 5: 561–576. 10.1111/ele.12757. 28317296

[gcb70407-bib-0070] Pershing, A. J. , M. A. Alexander , D. C. Brady , et al. 2021. “Climate Impacts on the Gulf of Maine Ecosystem: A Review of Observed and Expected Changes in 2050 From Rising Temperatures.” Elementa: Science of the Anthropocene 9, no. 1: e00076. 10.1525/elementa.2020.00076.

[gcb70407-bib-0071] Pershing, A. J. , M. A. Alexander , C. M. Hernandez , et al. 2015. “Slow Adaptation in the Face of Rapid Warming Leads to Collapse of the Gulf of Maine Cod Fishery.” Science 350, no. 6262: 809–812. 10.1126/science.aac9819. 26516197

[gcb70407-bib-0072] Pinsky, M. L. , R. L. Selden , and Z. J. Kitchel . 2020. “Climate‐Driven Shifts in Marine Species Ranges: Scaling From Organisms to Communities.” Annual Review of Marine Science 12, no. 1: 153–179. 10.1146/annurev-marine-010419-010916. 31505130

[gcb70407-bib-0073] Poloczanska, E. S. , C. J. Brown , W. J. Sydeman , et al. 2013. “Global Imprint of Climate Change on Marine Life.” Nature Climate Change 3, no. 10: 919–925. 10.1038/nclimate1958.

[gcb70407-bib-0074] Quattrini, A. M. , M. S. Nizinski , J. D. Chaytor , et al. 2015. “Exploration of the Canyon‐Incised Continental Margin of the Northeastern United States Reveals Dynamic Habitats and Diverse Communities.” PLoS One 10, no. 10: e0139904. 10.1371/journal.pone.0139904. 26509818 PMC4624883

[gcb70407-bib-0075] R Core Team . 2022. A Language and Environment for Statistical Computing. [Computer Software]. R Foundation for Statistical Computing. https://www.R‐project.org/.

[gcb70407-bib-0076] Rakka, M. , A. Metaxas , M. Nizinski , D. Packer , and M. Wall . 2025. “Ocean Circulation Drives Zonation of Deep‐Water Coral Communities and Their Traits in the Northwest Atlantic.” Progress in Oceanography 236: 103509. 10.1016/j.pocean.2025.103509.

[gcb70407-bib-0077] Ramirez‐Llodra, E. 2020. “Deep‐Sea Ecosystems: Biodiversity and Anthropogenic Impacts.” In The Law of the Seabed, 36–60. Brill Nijhoff. 10.1163/9789004391567_004.

[gcb70407-bib-0078] Roberts, S. M. , P. N. Halpin , and J. S. Clark . 2022. “Jointly Modeling Marine Species to Inform the Effects of Environmental Change on an Ecological Community in the Northwest Atlantic.” Scientific Reports 12, no. 1: 132. 10.1038/s41598-021-04110-0. 34997068 PMC8742080

[gcb70407-bib-0079] Roberts, S. M. , A.‐M. Jacoby , J. J. Roberts , et al. 2023. “Tight Spatial Coupling of a Marine Predator With Soniferous Fishes: Using Joint Modelling to Aid in Ecosystem Approaches to Management.” Diversity and Distributions 29, no. 8: 1074–1089. 10.1111/ddi.13746.

[gcb70407-bib-0080] Ryan, W. , S. Carbotte , J. Coplan , et al. 2009. “Global Multi‐Resolution Topography Synthesis.” Geochemistry, Geophysics, Geosystems 10: Q03014. 10.1029/2008GC002332.

[gcb70407-bib-0081] Saba, V. S. , S. M. Griffies , W. G. Anderson , et al. 2016. “Enhanced Warming of the Northwest Atlantic Ocean Under Climate Change.” Journal of Geophysical Research‐Oceans 121, no. 1: 118–132. 10.1002/2015JC011346.

[gcb70407-bib-0082] Santini, L. , A. Benítez‐López , L. Maiorano , M. Čengić , and M. A. J. Huijbregts . 2021. “Assessing the Reliability of Species Distribution Projections in Climate Change Research.” Diversity and Distributions 27, no. 6: 1035–1050. 10.1111/ddi.13252.

[gcb70407-bib-0083] Schneider, C. L. 2018. “Marine Refugia Past, Present, and Future: Lessons From Ancient Geologic Crises for Modern Marine Ecosystem Conservation.” In Marine Conservation Paleobiology, edited by C. L. Tyler and C. L. Schneider , 163–208. Springer International Publishing. 10.1007/978-3-319-73795-9_8.

[gcb70407-bib-0084] Sherwood, O. A. , and E. N. Edinger . 2009. “Ages and Growth Rates of Some Deep‐Sea Gorgonian and Antipatharian Corals of Newfoundland and Labrador.” Canadian Journal of Fisheries and Aquatic Sciences 66, no. 1: 142–152. 10.1139/F08-195.

[gcb70407-bib-0085] Siedlecki, S. , J. Salisbury , D. Gledhill , et al. 2021. “Projecting Ocean Acidification Impacts for the Gulf of Maine to 2050: New Tools and Expectations.” Elementa: Science of the Anthropocene 9, no. 1: e00062. 10.1525/elementa.2020.00062.

[gcb70407-bib-0086] Stanley, G. D. , H. M. E. Shepherd , and A. J. Robinson . 2018. “Paleoecological Response of Corals to the End‐Triassic Mass Extinction: An Integrational Analysis.” Journal of Earth Science 29, no. 4: 879–885. 10.1007/s12583-018-0793-5.

[gcb70407-bib-0087] Staudinger, M. D. , K. E. Mills , K. Stamieszkin , et al. 2019. “It's About Time: A Synthesis of Changing Phenology in the Gulf of Maine Ecosystem.” Fisheries Oceanography 28, no. 5: 532–566. 10.1111/fog.12429. 31598058 PMC6774335

[gcb70407-bib-0088] Stephenson, F. , D. A. Bowden , A. A. Rowden , et al. 2024. “Using Joint Species Distribution Modelling to Predict Distributions of Seafloor Taxa and Identify Vulnerable Marine Ecosystems in New Zealand Waters.” Biodiversity and Conservation 33, no. 11: 3103–3127. 10.1007/s10531-024-02904-y.

[gcb70407-bib-0089] Sweetman, A. K. , A. R. Thurber , C. R. Smith , et al. 2017. “Major Impacts of Climate Change on Deep‐Sea Benthic Ecosystems.” Elementa 5: 4. 10.1525/elementa.203.

[gcb70407-bib-0090] Takolander, A. , M. Cabeza , and E. Leskinen . 2017. “Climate Change Can Cause Complex Responses in Baltic Sea Macroalgae: A Systematic Review.” Journal of Sea Research 123: 16–29. 10.1016/j.seares.2017.03.007.

[gcb70407-bib-0091] Thurber, A. R. , A. K. Sweetman , B. E. Narayanaswamy , D. O. B. Jones , J. Ingels , and R. L. Hansman . 2014. “Ecosystem Function and Services Provided by the Deep Sea.” Biogeosciences 11, no. 14: 3941–3963. 10.5194/bg-11-3941-2014.

[gcb70407-bib-0092] Tikhonov, G. , Ø. H. Opedal , N. Abrego , et al. 2020. “Joint Species Distribution Modelling With the r‐Package Hmsc.” Methods in Ecology and Evolution 11, no. 3: 442–447. 10.1111/2041-210X.13345. 32194928 PMC7074067

[gcb70407-bib-0093] Townsend, D. W. , N. R. Pettigrew , M. A. Thomas , M. G. Neary , D. J. McGillicuddy , and J. O'Donnell . 2015. “Water Masses and Nutrient Sources to the Gulf of Maine.” Journal of Marine Research 73, no. 3–4: 93–122. 10.1357/002224015815848811. 27721519 PMC5051723

[gcb70407-bib-0094] van der Plas, F. 2019. “Biodiversity and Ecosystem Functioning in Naturally Assembled Communities.” Biological Reviews 94, no. 4: 1220–1245. 10.1111/brv.12499. 30724447

[gcb70407-bib-0095] van Gennip, S. J. , E. E. Popova , A. Yool , G. T. Pecl , A. J. Hobday , and C. J. B. Sorte . 2017. “Going With the Flow: The Role of Ocean Circulation in Global Marine Ecosystems Under a Changing Climate.” Global Change Biology 23, no. 7: 2602–2617. 10.1111/gcb.13586. 27935174

[gcb70407-bib-0096] Wang, S. , F. J. Murillo , and E. Kenchington . 2022. “Climate‐Change Refugia for the Bubblegum Coral *Paragorgia Arborea* in the Northwest Atlantic.” Frontiers in Marine Science 9: 863693. 10.3389/fmars.2022.863693.

[gcb70407-bib-0097] Wei, C.‐L. , J. M. González‐Irusta , C. Domínguez‐Carrió , and T. Morato . 2020. Set of Terrain (Static in Time) and Environmental (Dynamic in Time) Variables Used as Candidate Predictors of Present‐Day (1951‐2000) and Future (2081‐2100) Suitable Habitat of Cold‐Water Corals and Deep‐Sea Fishes in the North Atlantic [Dataset]. Pangaea. 10.1594/PANGAEA.911117.

[gcb70407-bib-0098] Worm, B. , and H. K. Lotze . 2021. “Chapter 21—Marine Biodiversity and Climate Change.” In Climate Change, edited by T. M. Letcher , Third ed., 445–464. Elsevier. 10.1016/B978-0-12-821575-3.00021-9.

[gcb70407-bib-0099] Wright, G. , K. M. Gjerde , D. E. Johnson , et al. 2021. “Marine Spatial Planning in Areas Beyond National Jurisdiction.” Marine Policy 132: 103384. 10.1016/j.marpol.2018.12.003.

[gcb70407-bib-0100] Xavier, J. R. , S. A. Pomponi , and E. L. Kenchington . 2023. “Editorial: Deep‐Sea Sponge Ecosystems: Knowledge‐Based Approach Towards Sustainable Management and Conservation.” Frontiers in Marine Science 10: 1132451. 10.3389/fmars.2023.1132451.

[gcb70407-bib-0101] Xu, T. , C. Zhang , B. Xu , Y. Xue , Y. Ji , and Y. Ren . 2025. “Spatial Random Effects Improve the Predictions of Multispecies Distribution in a Marine Fish Assemblage.” Journal of Ocean University of China 24, no. 2: 471–482. 10.1007/s11802-025-5965-1.

[gcb70407-bib-0102] Yasuhara, M. , and R. Danovaro . 2016. “Temperature Impacts on Deep‐Sea Biodiversity.” Biological Reviews 91, no. 2: 275–287. 10.1111/brv.12169. 25523624

[gcb70407-bib-0103] Zheng, M.‐D. , and L. Cao . 2014. “Simulation of Global Ocean Acidification and Chemical Habitats of Shallow‐ and Cold‐Water Coral Reefs.” Advances in Climate Change Research 5, no. 4: 189–196. 10.1016/j.accre.2015.05.002.

