# Supplementary material for: Climate Change Drives Bathymetric Shifts in Taxonomic and Trait Diversity of Deep‐Sea Benthic Communities
Source: Glob Chang Biol. 2025 Aug 5;31(8):e70407. doi: 10.1111/gcb.70407 (PMC12322877; doi:10.1111/gcb.70407)
Supplement: Supplementary file 1 — Data S1: gcb70407‐sup‐0001‐Supinfo1.pdf. [file GCB-31-e70407-s001.pdf]

Supplementary Information 1: Model development

Coral data

Coral data were compiled from nine research expeditions (surveys, Figure S1.1) that took place within the study area between 2013-2019. Expeditions used either Remotely Operated Vehicles (ROVs) or towed cameras and performed numerous dives. Data from these surveys were firstly used to construct the coral genus pool, i.e. the complete list of coral genera that were observed in the study area. Genera with less than five observations in the dataset were considered very rare and were excluded from the analysis, leading to a pool of 30 coral genera (Table S1.1). Subsequently, the survey data were used to infer presence of corals within each cell of the model grid (1 km resolution). Genera that were not observed within a cell were considered absent. Since the surveys did not cover the complete surface of each cell, absences are rather considered pseudoabsences.

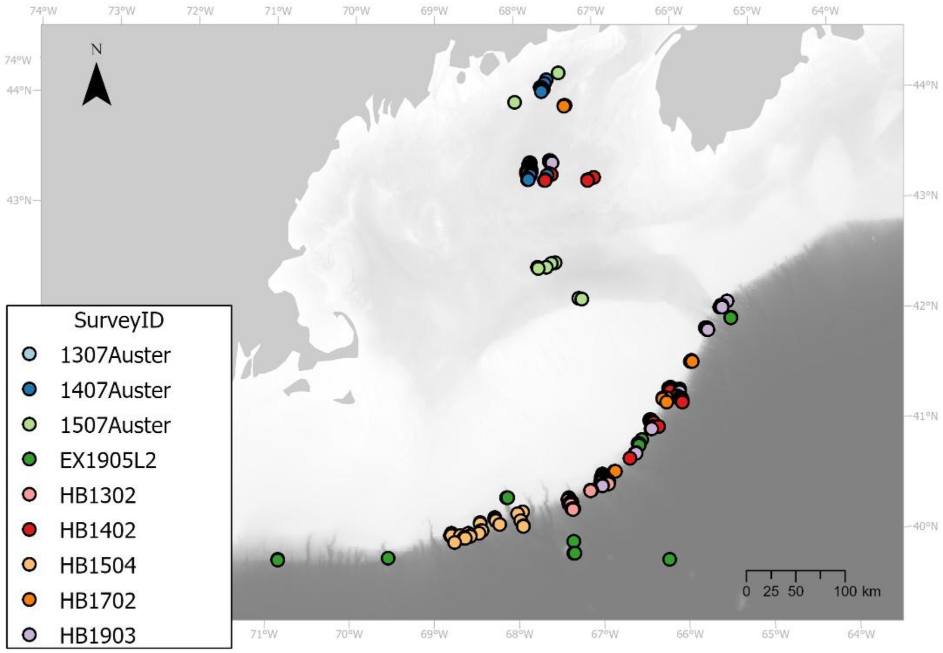

Figure S1.1: Locations of ROV and towed camera dives that were used to compile data on presence of 30 deep-water coral genera in the study area. Dives were performed during nine research expeditions (surveys).

Table S1.1: Coral genera included in the study and their taxonomic classification

Subclass	Order	Genus
Hexacorallia	Antipatharia	Bathypathes
		Parantipathes
		Stauropathes
		Stichopathes
		Telopathes
	Scleractinia	Desmophyllum
		Javania
		Lophelia
		Solenosmilia
Octocorallia	Malacalcyonacea	Paramuricea
		Acanthogorgia
		Anthothela
		Clavularia
		Lateothela
		Swiftia
	Scleralcyonacea	Acanella
		Anthomastus
		Balticina
		Chrysogorgia
		Distichoptilum
		Funiculina
		Keratoisis
		Kophobelemnon
		Paragorgia
		Pennatula
		Primnoa
		Protoptilum
		Radicipes
		Thouarella
		Umbellula

Environmental variables

In total, 21 environmental variables were examined for the development of the HMSC models. We used the Pearson correlation index and the Variance Inflation Factor (VIF) to exclude highly

correlated variables (i.e. those with Pearson>0.7, VIF>0.5). Additional information on variable sources and selection can be found at Rakka et al., (in review).

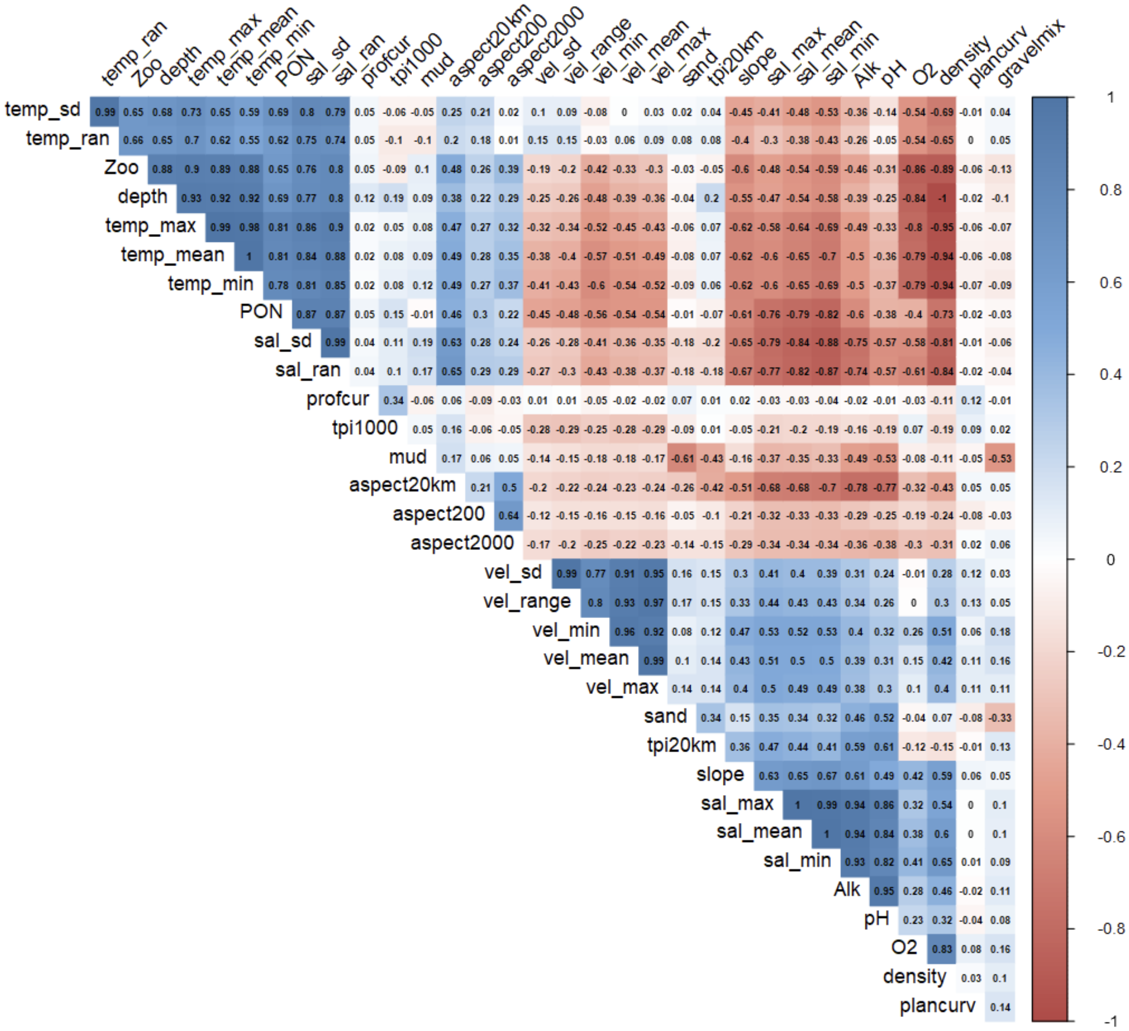

Figure S1.2: Correlogram showing values of Pearson correlation index between pairs of environmental variables. Zoo: surface zooplankton concentration, temp: bottom temperature, PON: surface particulate Organic Nitrogen, sal: bottom salinity, O2: bottom dissolved oxygen concentration, vel: bottom current velocity, density: bottom water density, pH: bottom pH, Alk: bottom alkalinity, profcurv: profile curvature, plancurv: plan curvature, tpi: topographic information index, mud: mud content, sand: sand content, gravelmix: gravel content. For bottom variables, the mean, min, max, standard deviation (sd) and range were considered. For terrain variables, the number next to the variable name indicates resolution in meters, unless otherwise indicated (e.g. tpi20km).

Table S1.2: Selected environmental variables used in the development of the Hierarchical Model of Species Communities.

Variable	Resolution	Source
Aspect	1 km	Calculated from bathymetry data (Global Multi-Resolution Topography, Ryan et al., 2009) by using the function *terrain* of the R package *raster*
Topographic Position Index	20 km	Calculated from bathymetry data (Global Multi-Resolution Topography, Ryan et al., 2009) by using the function *terrain* of the R package *raster*
Sediment type-mud percentage	1 km	Calculated by using kriging methods from data provided by the United States Geological Survey
Bottom temperature (average for 20)	1-1.5 km	FVCOM-GoM/GB v3
Bottom salinity	1-1.5 km	FVCOM-GoM/GB v3
Bottom current velocity	1-1.5 km	FVCOM-GoM/GB v3, calculated as: sqrt(Velocity eastward^2 + Velocity northward^2 + Velocity upward^2)

Ryan, W.B.F., S.M. Carbotte, J.O. Coplan, S. O'Hara, A. Melkonian, R. Arko, R.A. Weissel, V. Ferrini, A. Goodwillie, F. Nitsche, J. Bonczkowski, and R. Zemsky (2009), Global Multi-Resolution Topography synthesis, Geochem. Geophys. Geosyst., 10, Q03014, doi: 10.1029/2008GC002332

Coral traits

Initially we considered 21 coral traits, including life history traits (e.g. fecundity, oocyte size, growth) and morphological traits (e.g. colony surface, surface to volume ratio, colony shape). Most of these traits were excluded due to lack of data. As a result, we only used 3 morphological traits (Table S1.3). Polyp diameter and colony height were log-transformed to obtain a normal distribution. Skeletal material was expressed as a fuzzy variable with three categories: aragonite (%), calcite (%) and scleroprotein (%). For Scleractinia, we considered that their skeleton is composed of 100% aragonite. For Octocorallia, we assumed that the skeletal axis contributes 70% of the skeletal material and sclerites contribute 30%. For genera of octocorals that can grow without a central axis, we assumed that sclerites contribute 100% to the skeletal material.

Table S1.3: Coral traits used for the development of the HMSC models

Trait	Type	Trait categories	Unit	Sources
Polyp diameter	Numeric average		mm	Survey images, collections, taxonomic descriptions
Colony height	Numeric Average		mm	Survey images, collections, taxonomic descriptions, literature
Skeletal material	Fuzzy variable	Aragonite	%	Taxonomic descriptions, literature
		Calcite	%	Taxonomic descriptions, literature
		Scleroprotein	%	Taxonomic descriptions, literature

Table S1.4: Number of specimens and additional literature resources used for determining traits for the 30 coral genera used in the study.

Genus	Number of specimens	Additional literature resources
Acanella	24	3
Acanthogorgia	25	3
Anthomastus	4	2
Anthothela	11	3
Balticina	3	2
Bathypathes	4	4
Chrysogorgia	11	7
Clavularia	4	2
Desmophyllum	3	2
Distichoptilum	2	3
Funiculina	3	2
Javania	1	2
Keratoisis	17	2
Kophobelemnon	3	2
Lateothela	5	1
Lophelia	8	2
Paragorgia	19	4
Paramuricea	22	2
Parantipathes	1	5
Pennatula	12	2
Primnoa	17	4
Protoptilum	2	2
Radicipes	3	1
Solenosmilia	4	2
Stauropathes	3	3
Stichopathes	4	4
Swiftia	9	2
Telopathes	6	4
Thouarella	12	4
Umbellula	9	4

Trait-based approaches require the construction of the trait-space which is a three-dimensional space comprised of information for all traits. To construct the trait-space, we performed a Principal Coordinate Analysis based on the Gower distance, following Magneville et al., (2022).

We retained the first three PCoA axes to describe the trait space, as these explained most of the variance (20%, 15.3% and 7% for the three axes, respectively, 42% in total).

The first axis (PC1) was better described by a gradient from corals that utilize calcite, have large colony size and small polyp size, to corals that utilize aragonite, have small colony and large polyp size. The second axis (PC2) was better described by a gradient developing from corals that use calcite and have small colonies, to species that use scleroprotein and have large colonies. Lastly, the third axis (PC3) was better described by a gradient from corals with large colonies and large polyps, to corals with small colonies and small polyps.

Model Convergence and outputs

Table S1.5: Summary statistics of the Gelman–Rubin convergence index ($\hat{R}$) for all components of the Hierarchical Model of Species Communities (Beta: environmental variables, Gamma: traits, Omega: random effects at the level of the Dive and the grid cell). This index is used to assess the convergence of Markov Chain Monte Carlo (MCMC) simulations in Bayesian modeling. Values of $\hat{R} \approx 1$ suggest satisfactory convergence.

	Beta	Gamma	Omega-Dive	Omega-grid cell
**Min**	0.9978	0.998	0.9977	0.9981
**Max**	1.0575	1.050	1.0406	1.0219
**Mean**	1.0062	1.006	1.0061	1.0048
**Median**	1.0028	1.002	1.0045	1.0039
**1st quantile**	1.0008	1.000	1.0018	1.0013
**3rd quantile**	1.0063	1.007	1.0081	1.0070

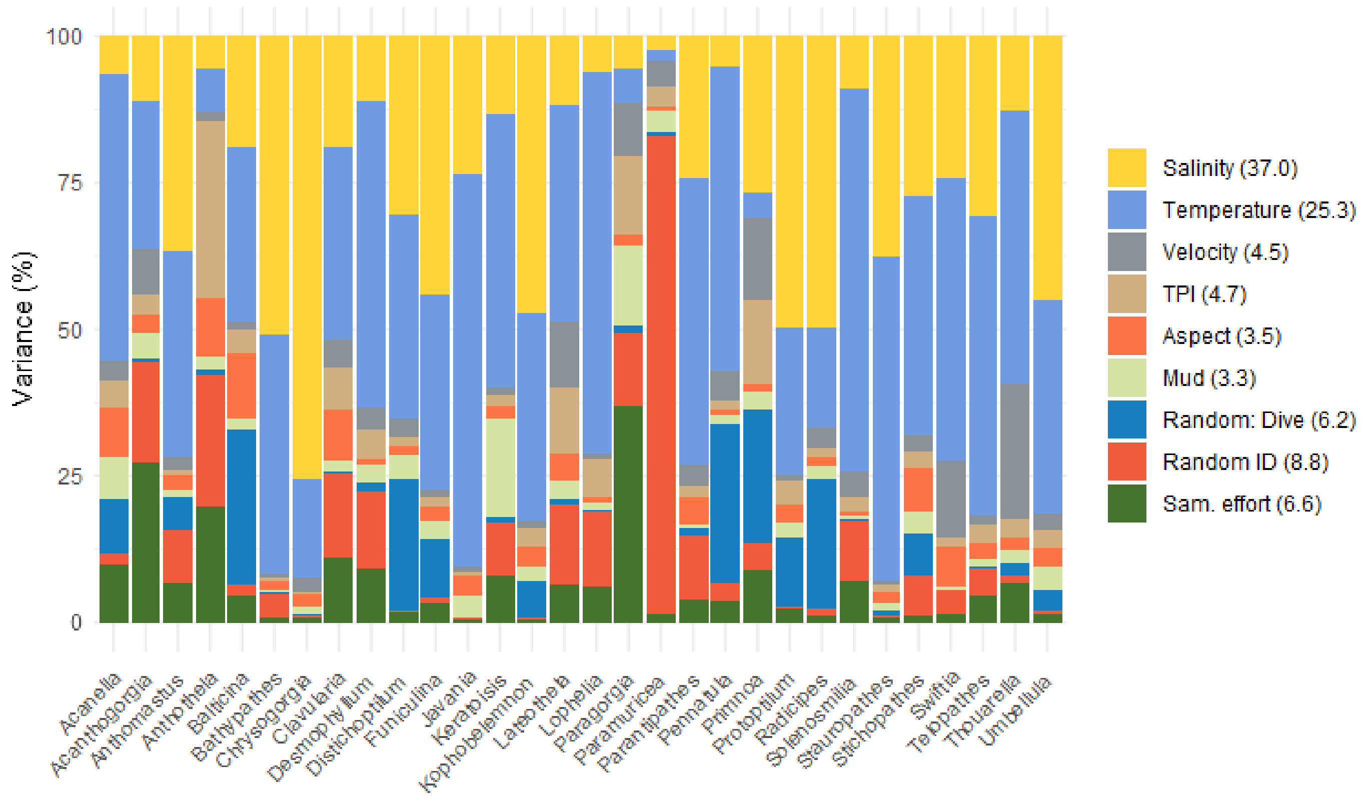

Figure S1.3: Variance partitioning among main variables for each of the coral genera included in the study.

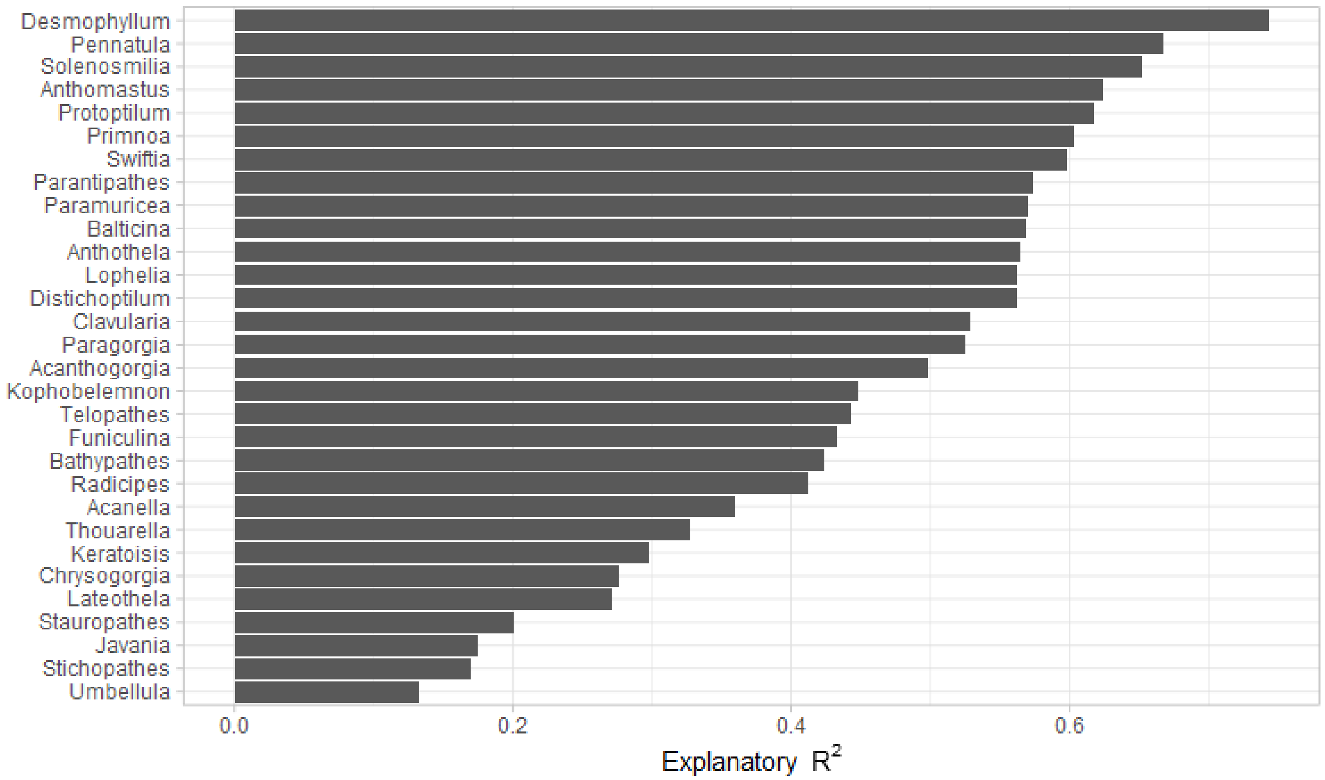

Figure S1.4: Explanatory power (R^2) of the constructed model for each coral genus. Genera are ordered to decreasing R^2.

Cross-validation

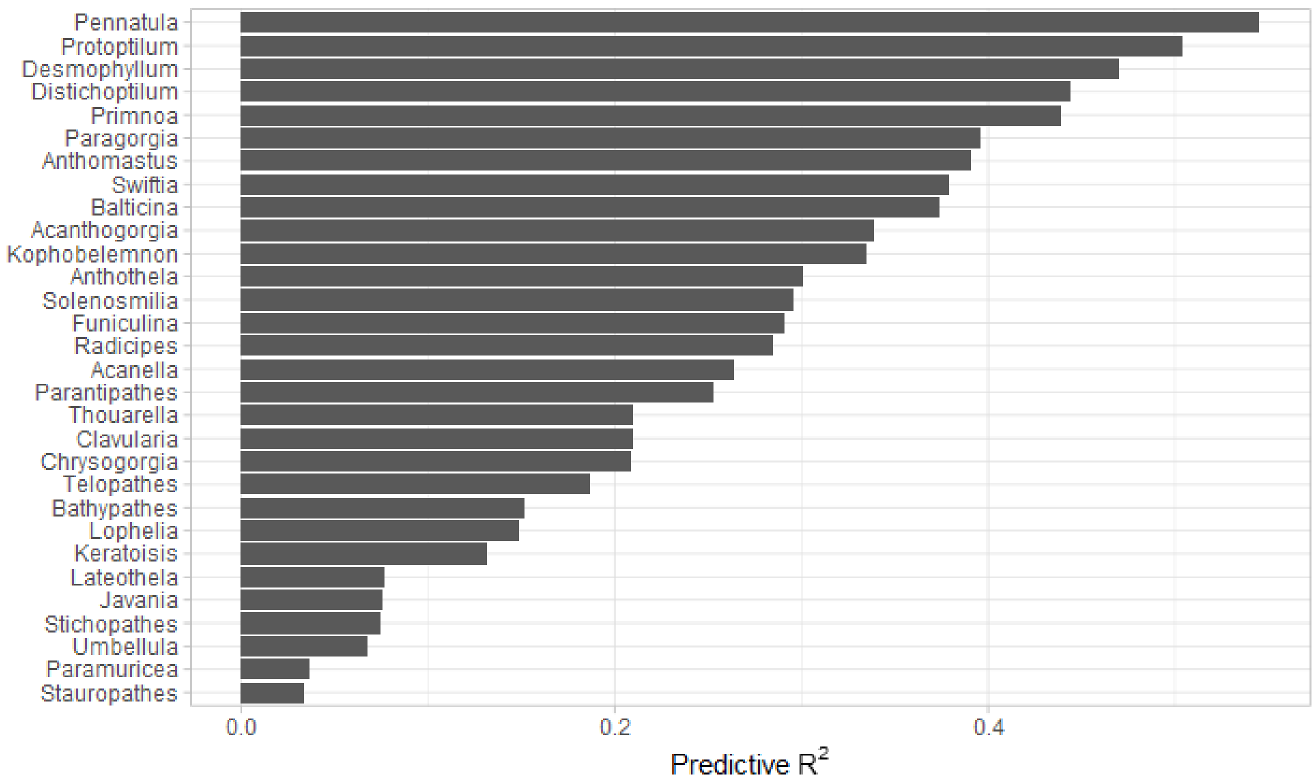

Figure S1.5: Predictive power (R^2) of the constructed model for each coral genus. Genera are ordered to decreasing R^2.

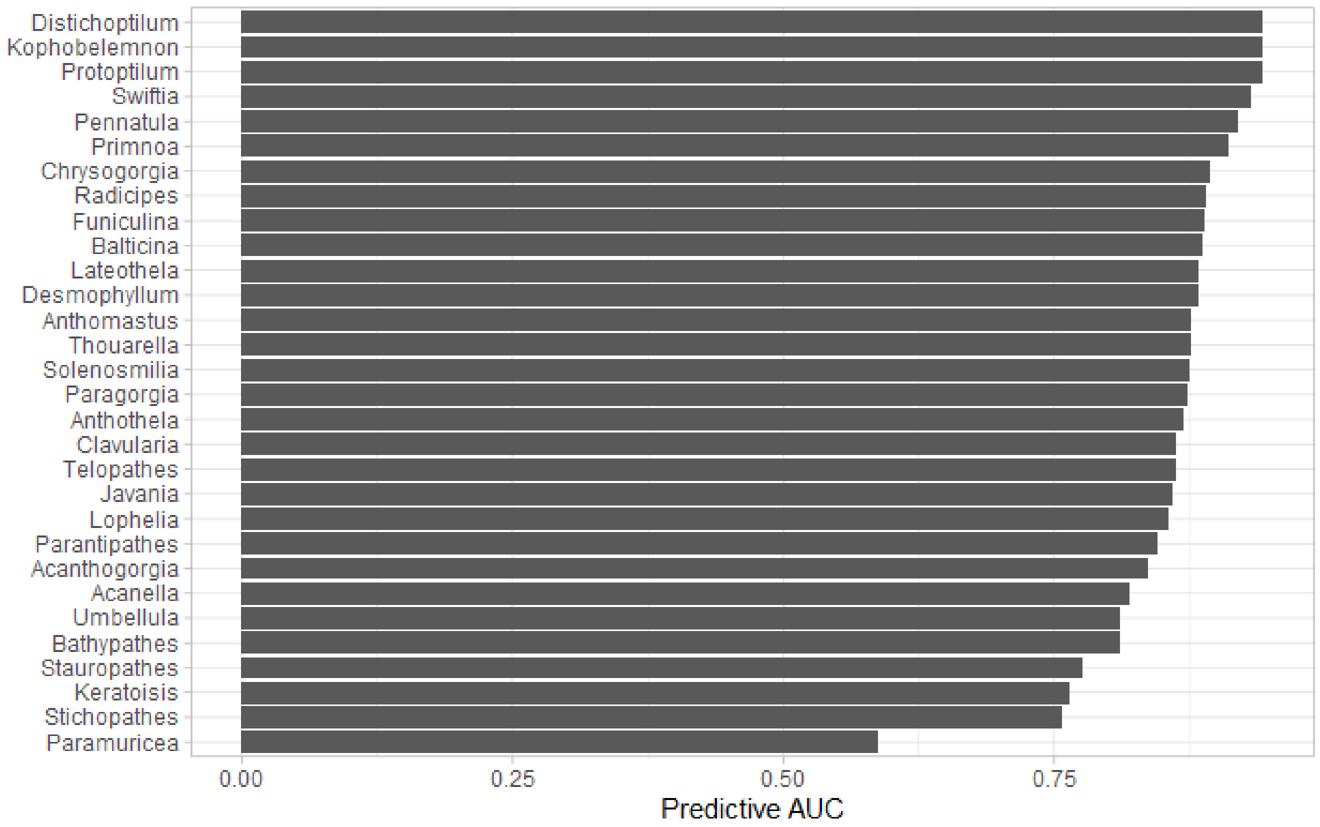

Figure S1.6: Predictive power (AUC) of the constructed model for each coral genus. Genera are ordered to decreasing AUC.

References

Magneville C, Loiseau N, Albouy C, Casajus N, Claverie T, Escalas A, Leprieur F, Maire E, Mouillot D, Villéger S (2022) mFD: an R package to compute and illustrate the multiple facets of functional diversity. Ecography. doi: 10.1111/ecog.05904
