# Supplementary material for: Climate Change Drives Bathymetric Shifts in Taxonomic and Trait Diversity of Deep‐Sea Benthic Communities
Source: Glob Chang Biol. 2025 Aug 5;31(8):e70407. doi: 10.1111/gcb.70407 (PMC12322877; doi:10.1111/gcb.70407)
Supplement: Supplementary file 2 — Data S2: gcb70407‐sup‐0002‐Supinfo2.pdf. [file GCB-31-e70407-s002.pdf]

Supplementary Information 2: Environmental Projections

Future Projections

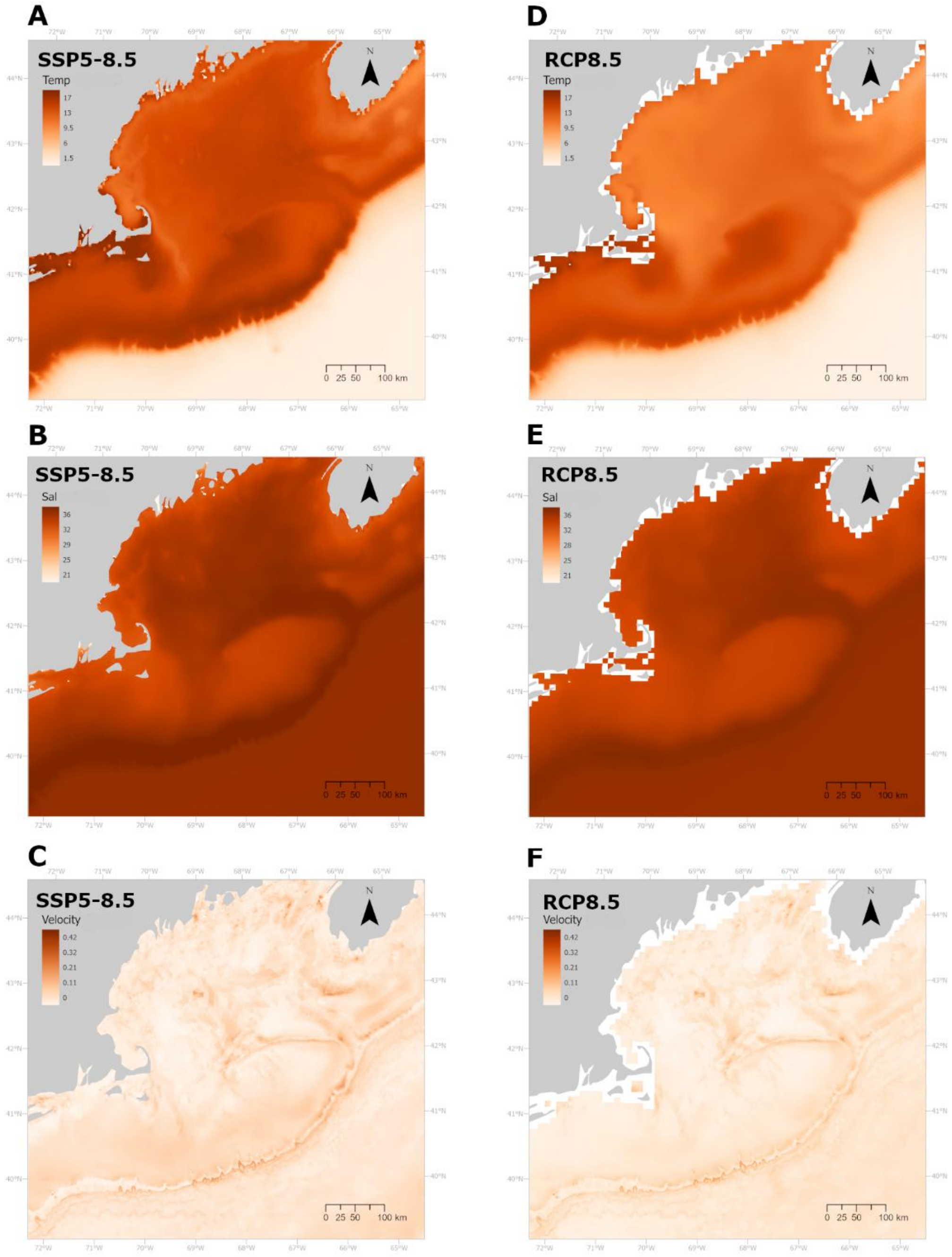

Figure S2.1: Future projections for Temperature (Temp, °C), Salinity (Sal), Velocity (Current, m/s) for the end of the century under two scenarios, SSP5-8.5 obtained from Bio-ORACLE (Assis et al., 2024) and RCP8.5 (Alexander et al., 2020).

Spatial extrapolation

Table S2.1: Results of the spatial extrapolation for environmental data of future climate projections for the end of the century under two scenarios, SSP5-8.5 obtained from Bio-ORACLE (Assis et al., 2024) and RCP8.5 (Alexander et al., 2020). Non-analogous cells represent those cells in the model grid with environmental data that exceed the data that were used to train the HMSC model.

Model	Type	Type	Covariate	Count	Percentage
**SSP5-8.5**	Non-analogous	Univariate	Temperature	469967	46
			Salinity	5851	5.7
			Velocity	38	0.037
			Aspect	14	0.014
			Total	52870	51.6
		Combinatorial	Aspect	93	0.091
			Mud	35	0.034
			Velocity	8	0.007
			Temperature	6	0.005
			Total		0.14
		Total		53012	51.74
	Analogous	Total		49442	48.26
**RCP8.5**	Non-analogous	Univariate	Temperature	45371	44
			Salinity	7604	7.4
			Aspect	13	0.013
			Total	52988	52
		Combinatorial	Mud	36	0.3
			Velocity	15	0.11
			Aspect	6	0.6
			Temperature	4	0.03
			Total	61	0.5
		Total		53049	51.78
	Analogous	Total		49405	48.22

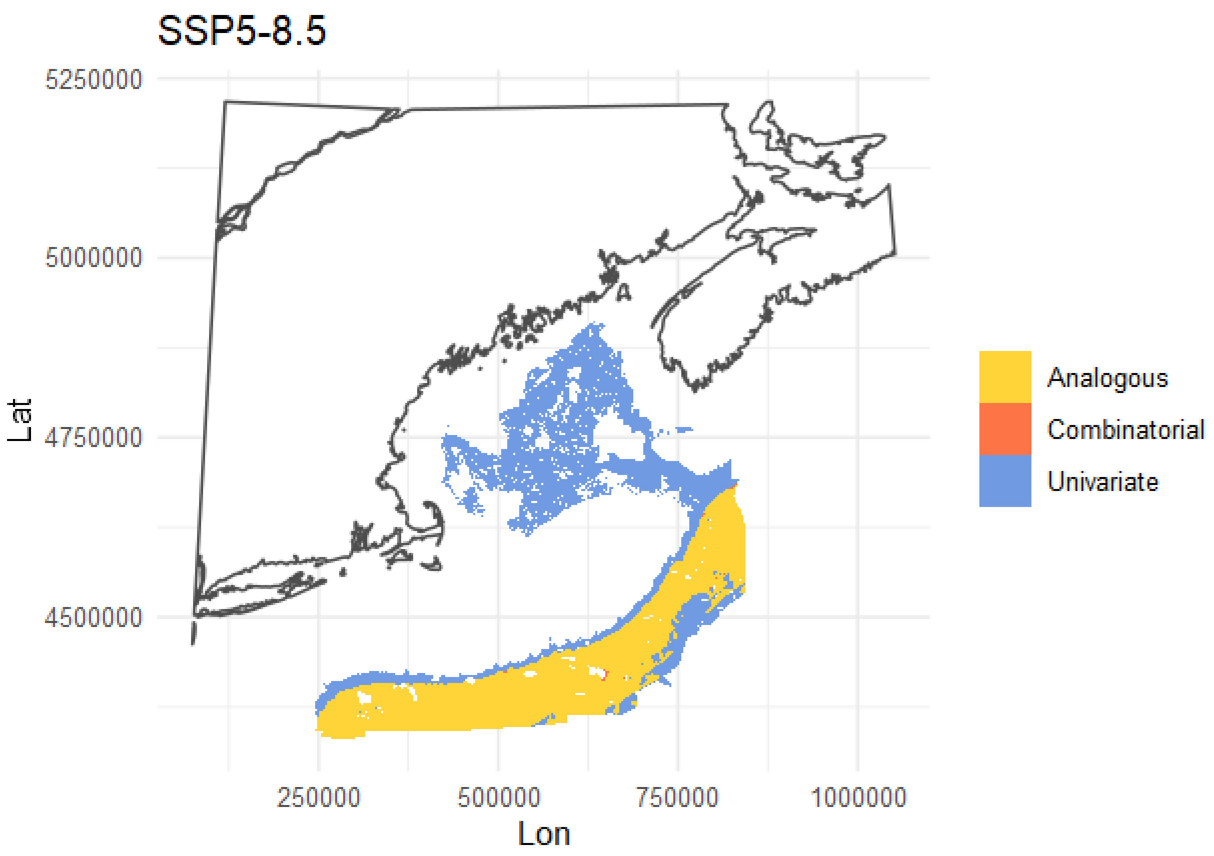

Figure S2.2: Results of the spatial extrapolation for environmental data of future climate projections for the end of the century under the SSP5-8.5 obtained from Bio-ORACLE (Assis et al., 2024). The map includes positions for analogous cells and non-analogous (combinatorial and univariate) cells. Non-analogous are those cells in the model grid with environmental data that exceed the data that were used to train the HMSC model. In univariate cells, the non-analogous conditions are due to a single variable, while in combinatorial conditions the combination of two variables was not included in the model.

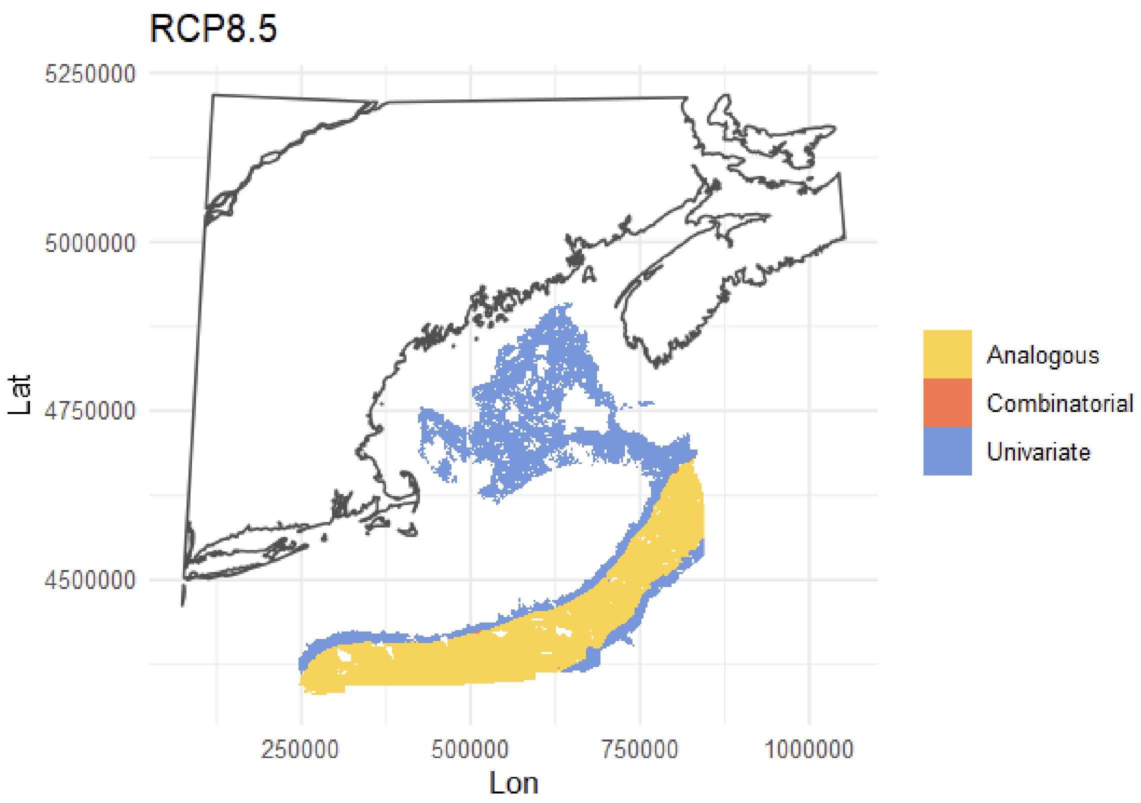

Figure S2.3: Results of the spatial extrapolation for environmental data of future climate projections for the end of the century under the RCP8.5 obtained from Alexander et al., (2020). The map includes positions for analogous cells and non-analogous (combinatorial and univariate) cells. Non-analogous are those cells in the model grid with environmental data that exceed the data that were used to train the HMSC model. In univariate cells, the non-analogous conditions are due to a single variable, while in combinatorial conditions the combination of two variables was not included in the model.
