# Supplementary material for: Climate Change Drives Bathymetric Shifts in Taxonomic and Trait Diversity of Deep‐Sea Benthic Communities
Source: Glob Chang Biol. 2025 Aug 5;31(8):e70407. doi: 10.1111/gcb.70407 (PMC12322877; doi:10.1111/gcb.70407)
Supplement: Supplementary file 3 — Data S3: gcb70407‐sup‐0003‐Supinfo3.pdf. [file GCB-31-e70407-s003.pdf]

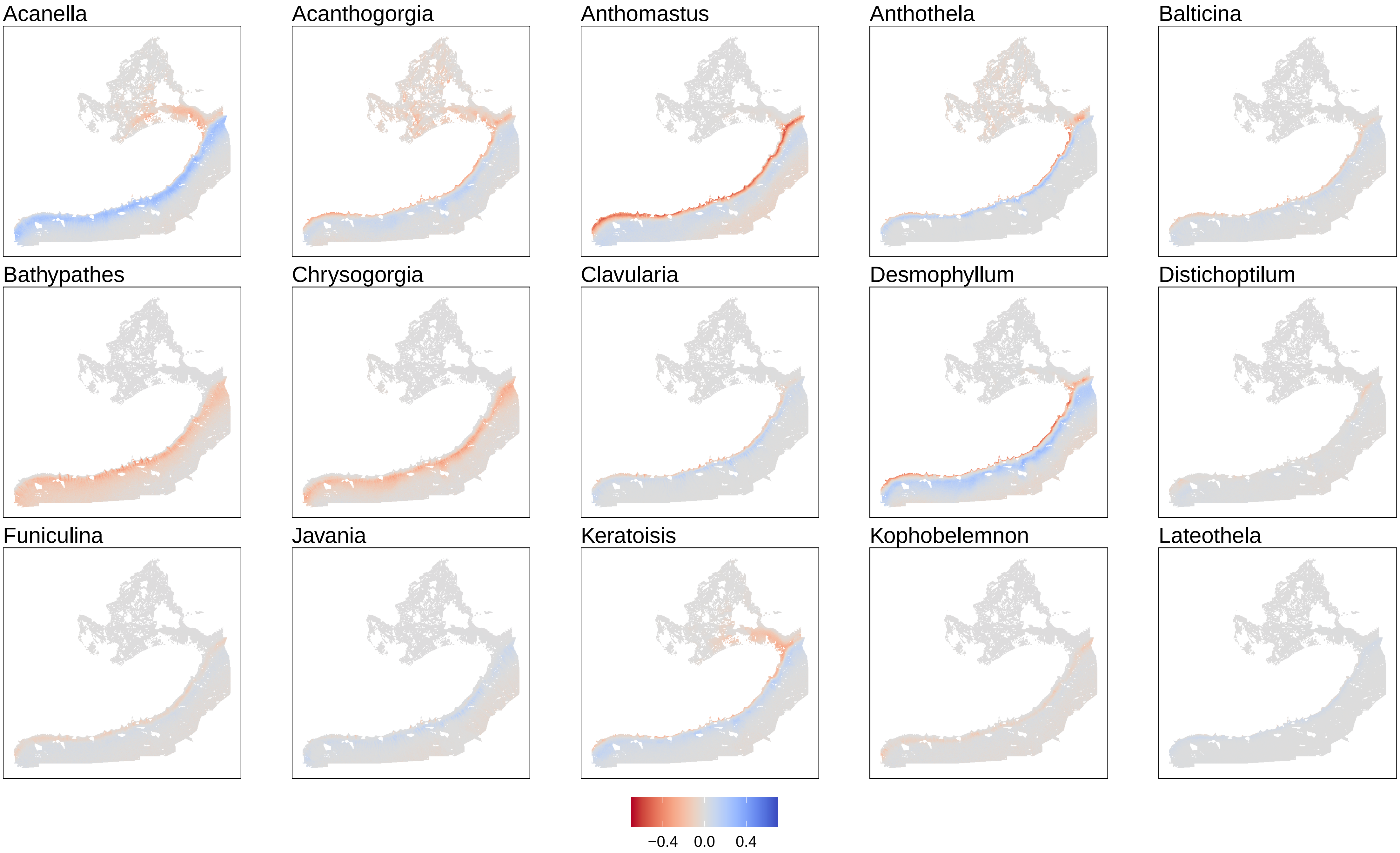

Figure S3.1: Delta values of the probability of occurrence of 30 deep-water coral genera under the RCP8.5 climate scenario for 2100.

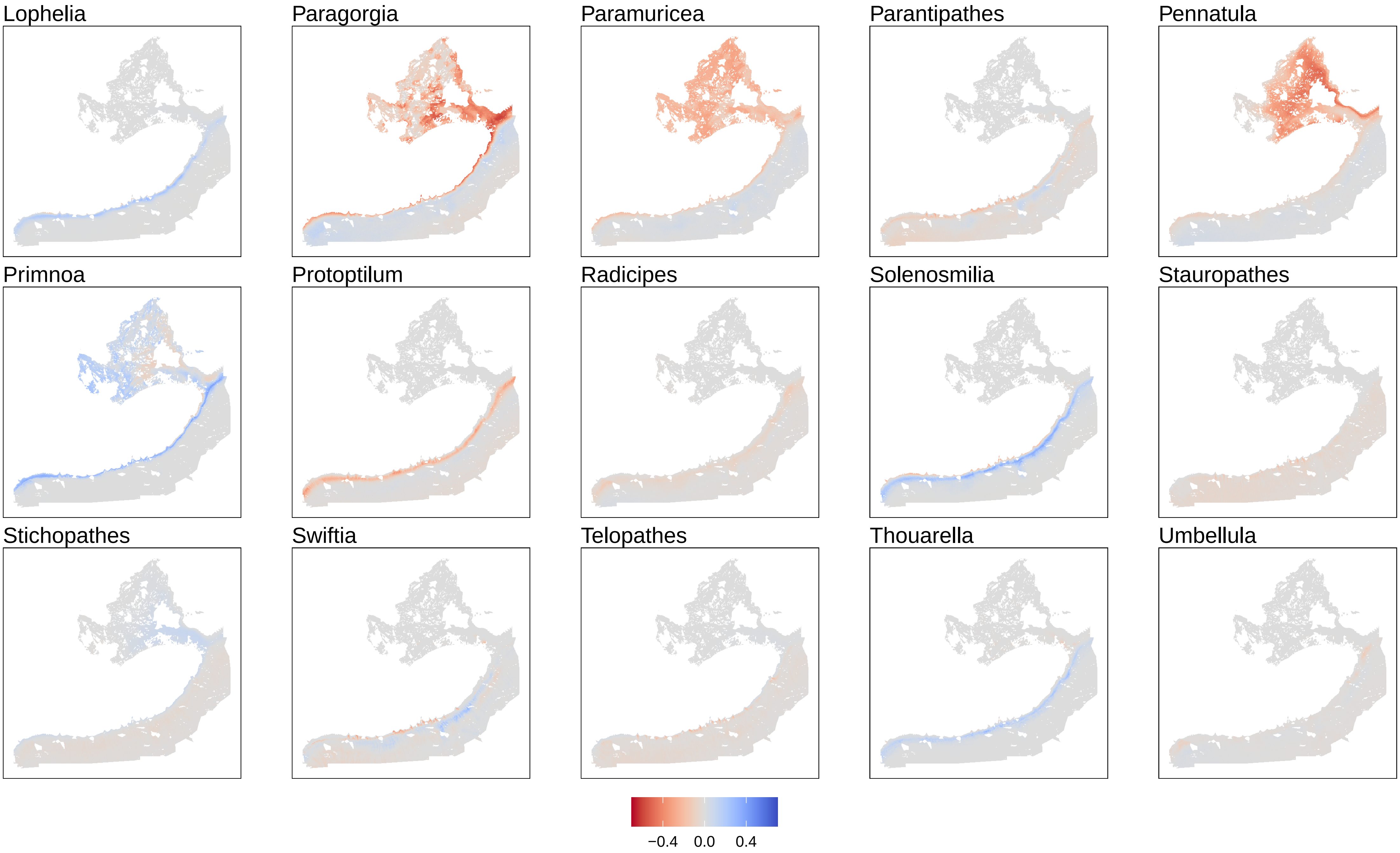

Figure S3.1 (cont): Delta values of the probability of occurrence of 30 deep-water coral genera under the RCP8.5 climate scenario for 2100.
