# Supplementary material for: Climate Change Drives Bathymetric Shifts in Taxonomic and Trait Diversity of Deep‐Sea Benthic Communities
Source: Glob Chang Biol. 2025 Aug 5;31(8):e70407. doi: 10.1111/gcb.70407 (PMC12322877; doi:10.1111/gcb.70407)
Supplement: Supplementary file 5 — Data S5: gcb70407‐sup‐0005‐Supinfo5.pdf. [file GCB-31-e70407-s005.pdf]

Supplementary Material 5: Evaluating uncertainty

Species richness

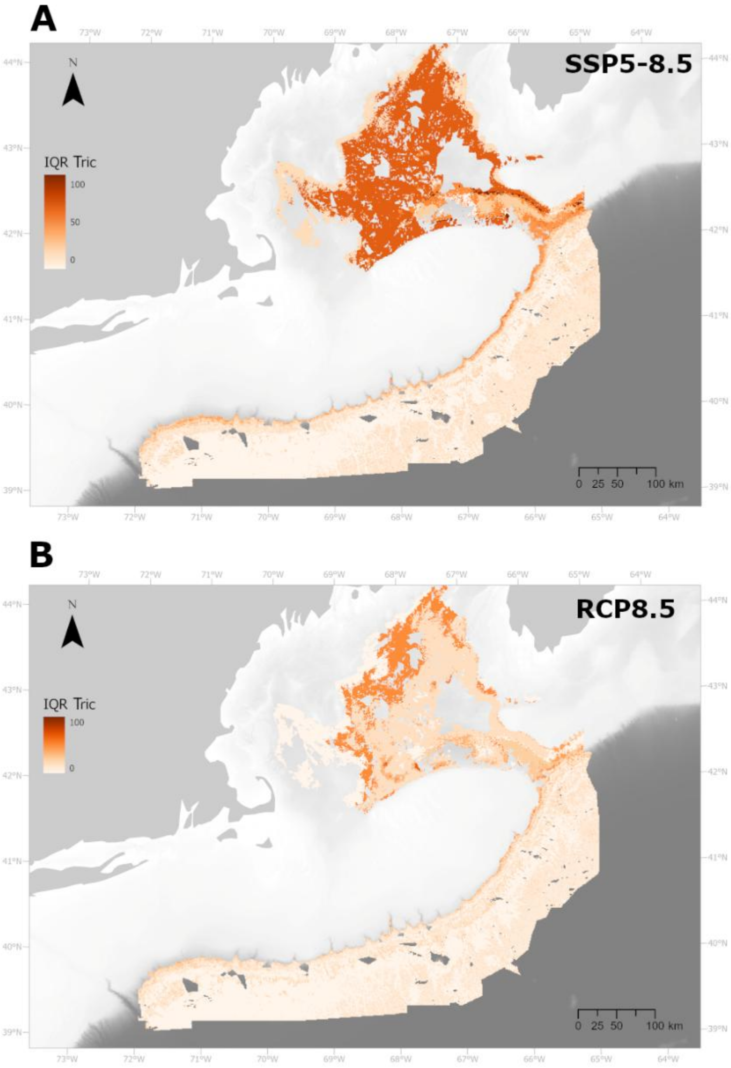

Figure S5.1. Interquantile range (range between the 25th and 75th percentile) for predictions of delta values of Taxonomic richness characterizing deep-water coral communities under the SSP5-8.5 (A), and RCP5.8 climate scenarios (B). Delta values represent differences between present and future conditions under the respective projection.

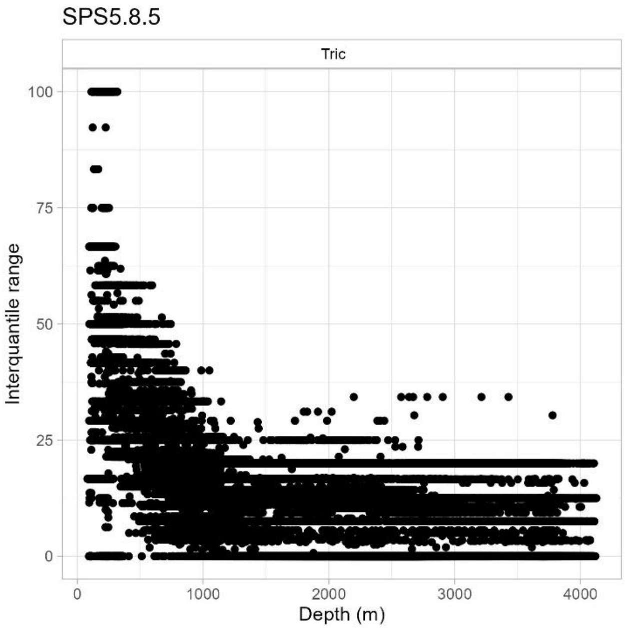

Figure S5.2: Interquantile range (range between the 25th and 75th percentile) of delta values of Taxonomic richness characterizing deep-water coral communities versus depth for predictions under the SSP5-8.5 climate scenario. Delta values represent differences between present and future conditions.

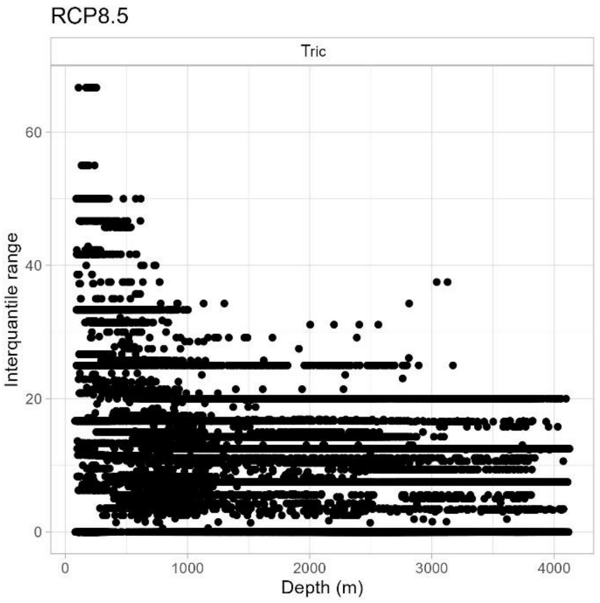

Figure S5.3: Interquantile range (range between the 25th and 75th percentile) of Taxonomic richness characterizing deep-water coral communities versus depth for predictions of delta values under the RCP8.5 climate scenario. Delta values represent differences between present and future conditions.

Trait diversity indices

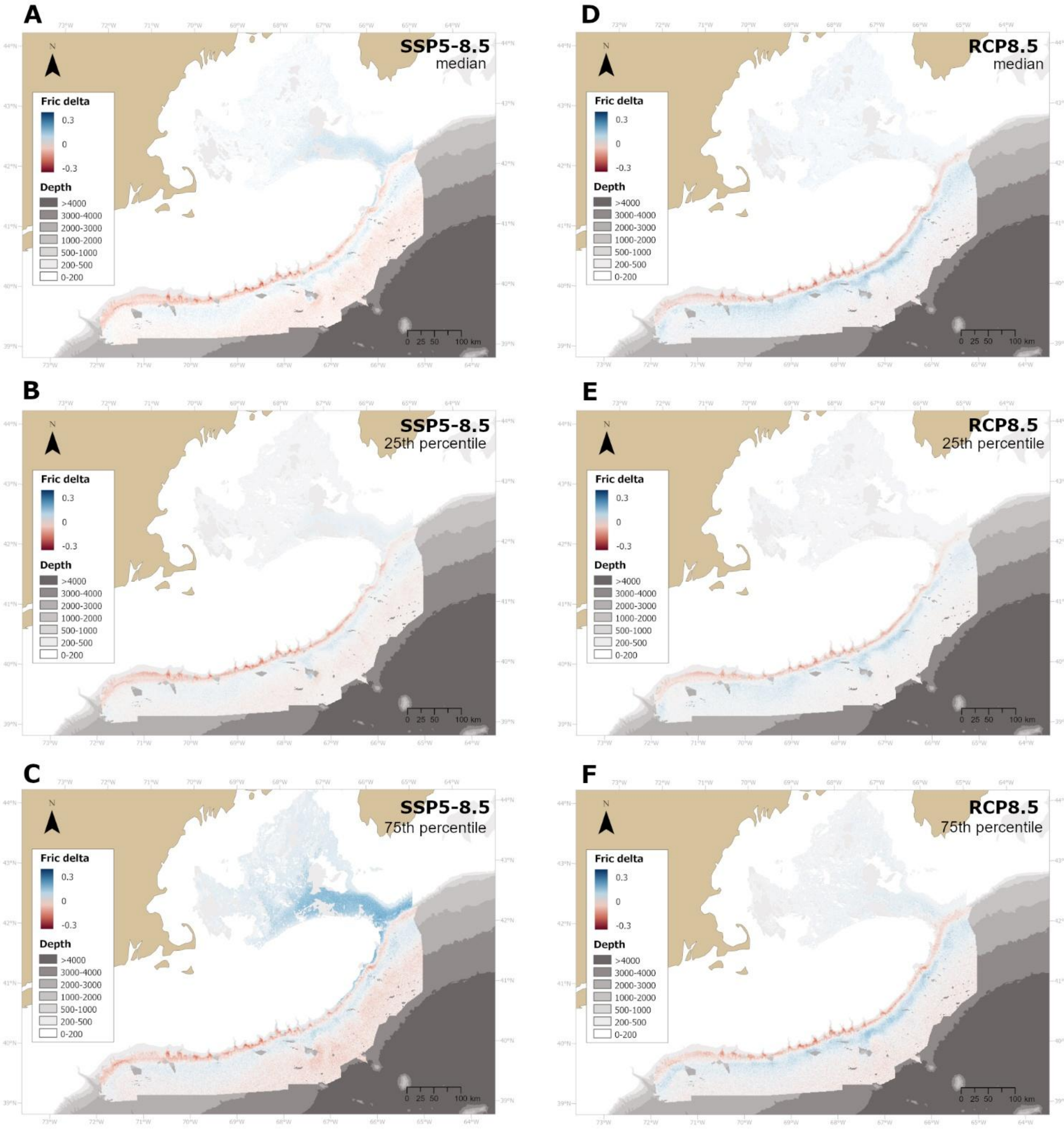

Figure S5.4: Delta values of Functional richness (Fric) characterizing deep-water coral communities under the SSP5-8.5 (A, B, C) and RCP5.8 (D, E, F) climate scenarios. Delta values represent differences between present and future conditions under the respective projection. Top figures (A, D) represent the median, middle figures (B,E) the 25th percentile, and bottom figures the 75th percentile.

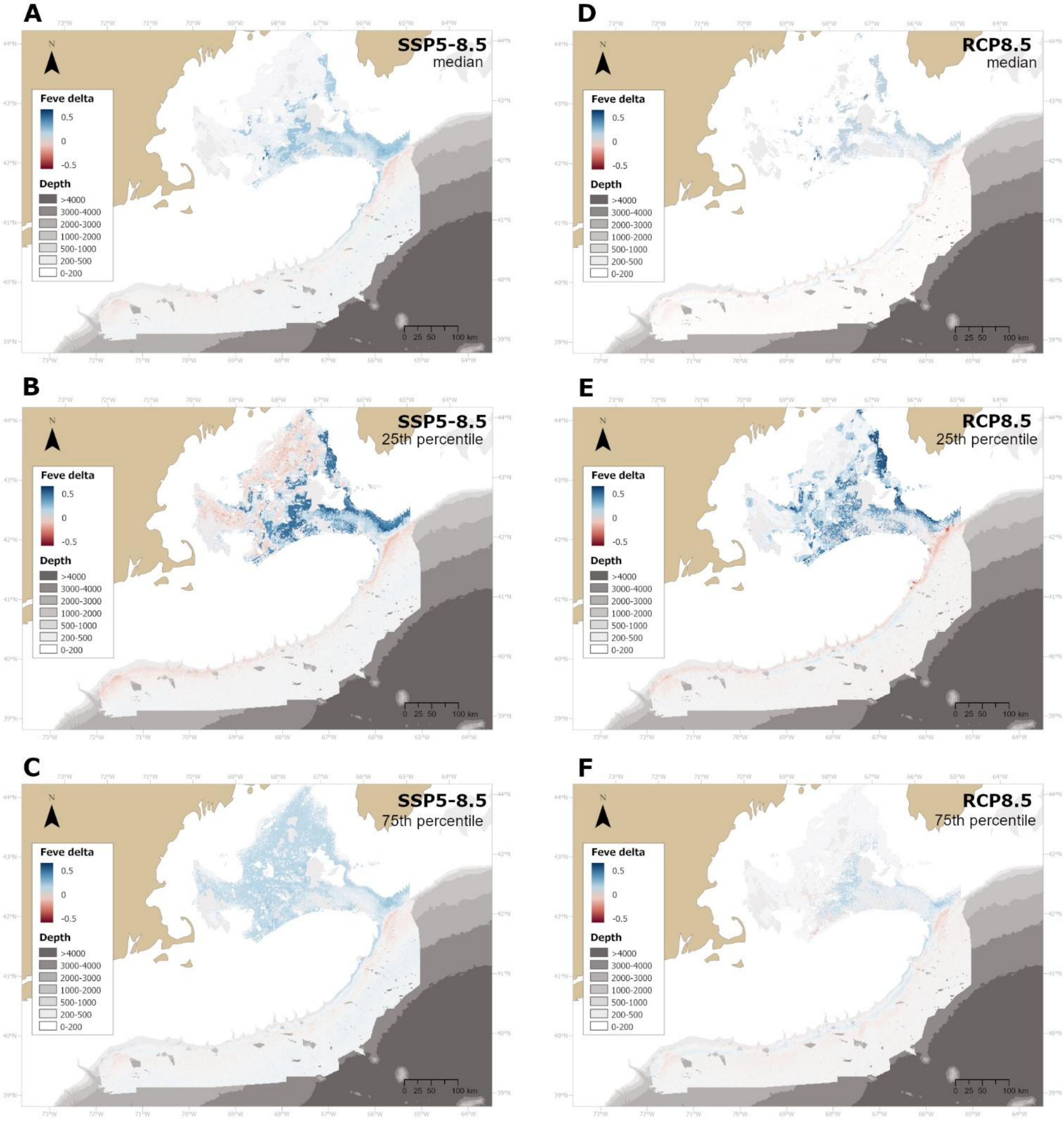

Figure S5.5: Delta values of Functional evenness (Feve) characterizing deep-water coral communities under the SSP5-8.5 (A, B, C) and RCP5.8 (D, E, F) climate scenarios. Delta values represent differences between present and future conditions under the respective projection. Top figures (A, D) represent the median, middle figures (B,E) the 25th percentile, and bottom figures the 75th percentile.

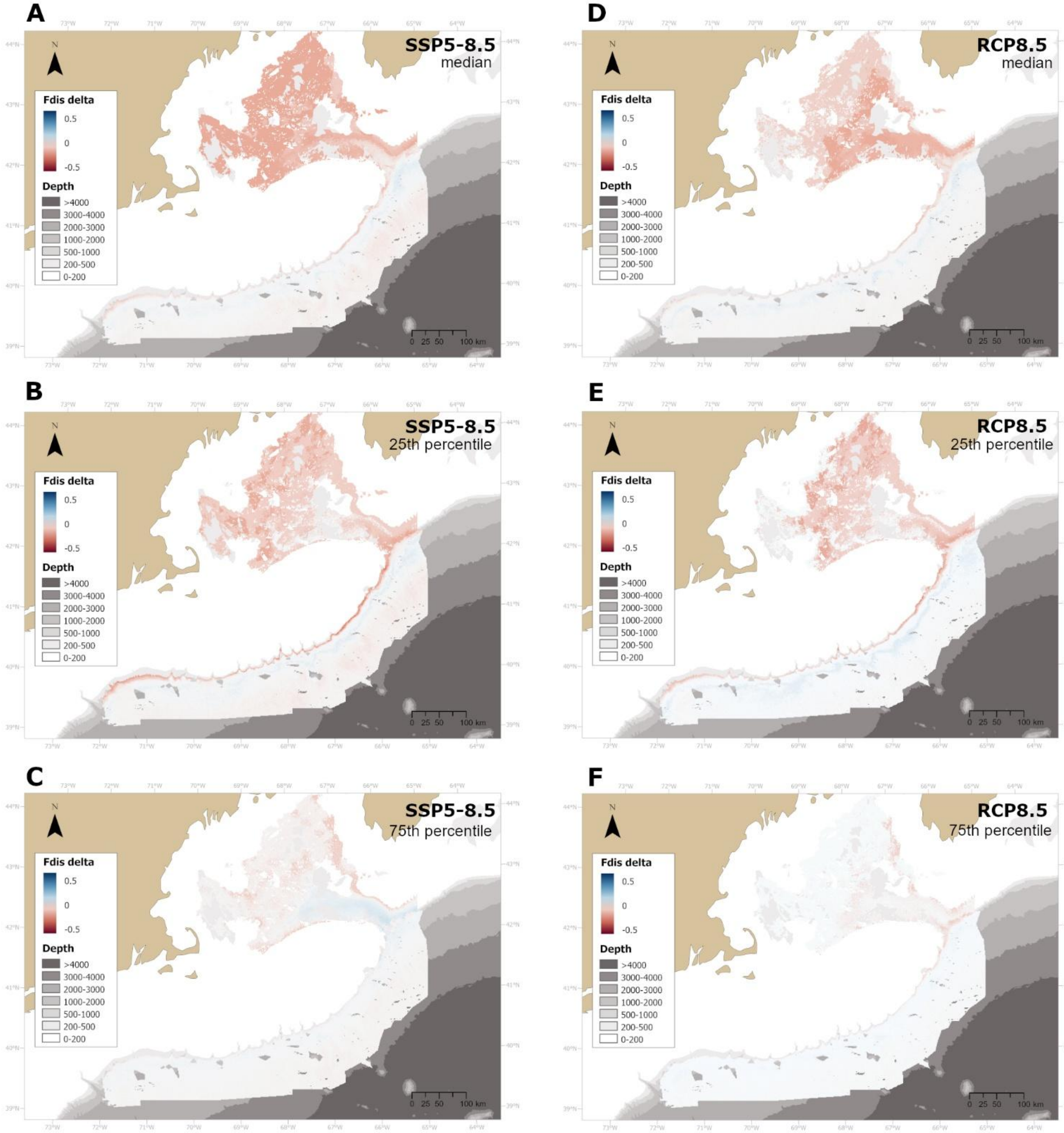

Figure S5.6: Delta values of Functional dispersion (Fdis) characterizing deep-water coral communities under the SSP5-8.5 (A, B, C) and RCP5.8 (D, E, F) climate scenarios. Delta values represent differences between present and future conditions under the respective projection. Top figures (A, D) represent the median, middle figures (B,E) the 25th percentile, and bottom figures the 75th percentile.

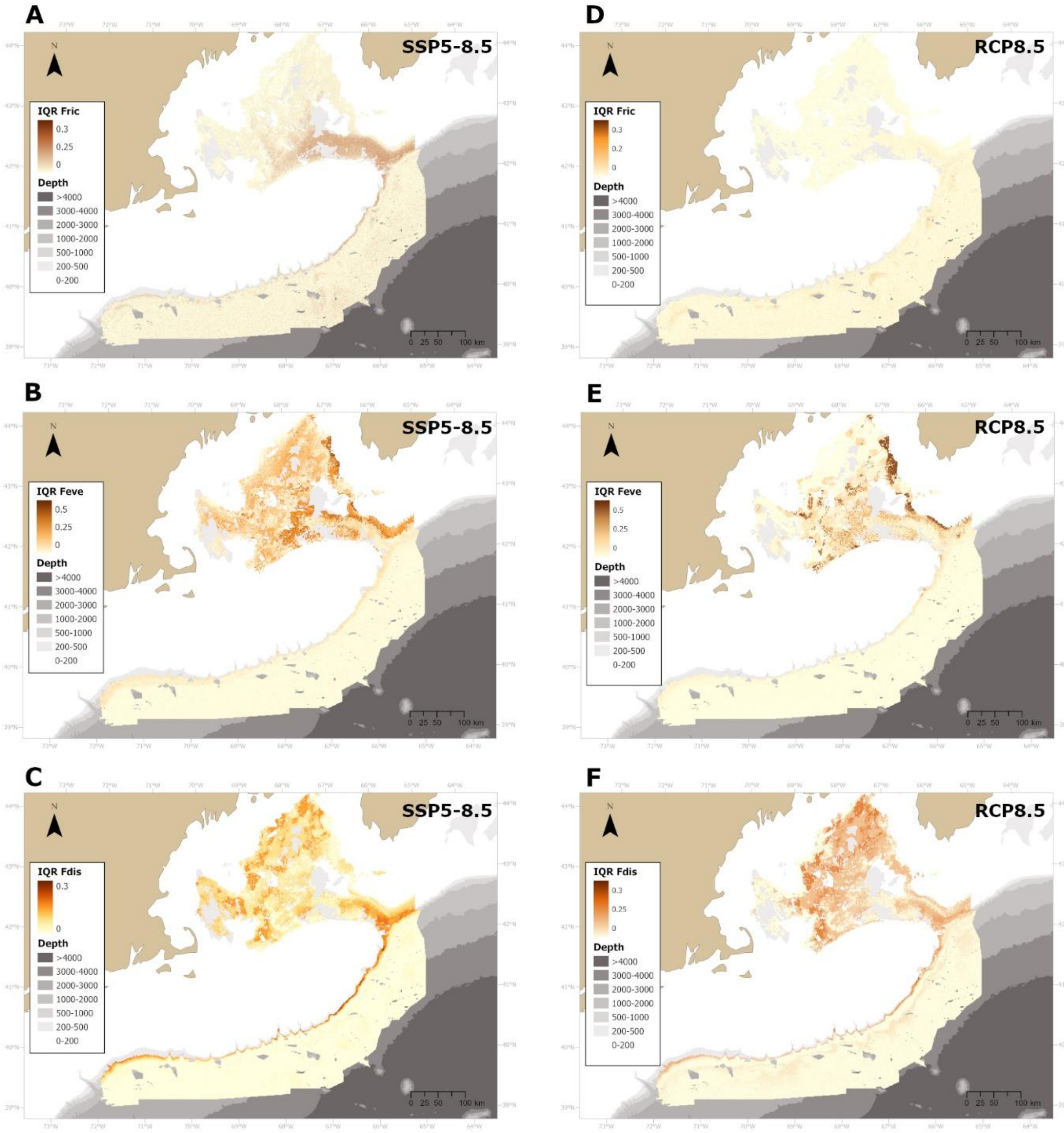

Figure S5.7*:* Interquantile range (range between the 25th and 75th percentile) for predictions of delta values of three trait diversity indices characterizing deep-water coral communities under the SPS5.8.5 (A, B, C) and RCP5.8 (D, E, F) climate scenarios. Delta values represent differences between present and future conditions under the respective projection. Fric: Functional richness, Feve: Functional evenness, Fdis: Functional dispersion

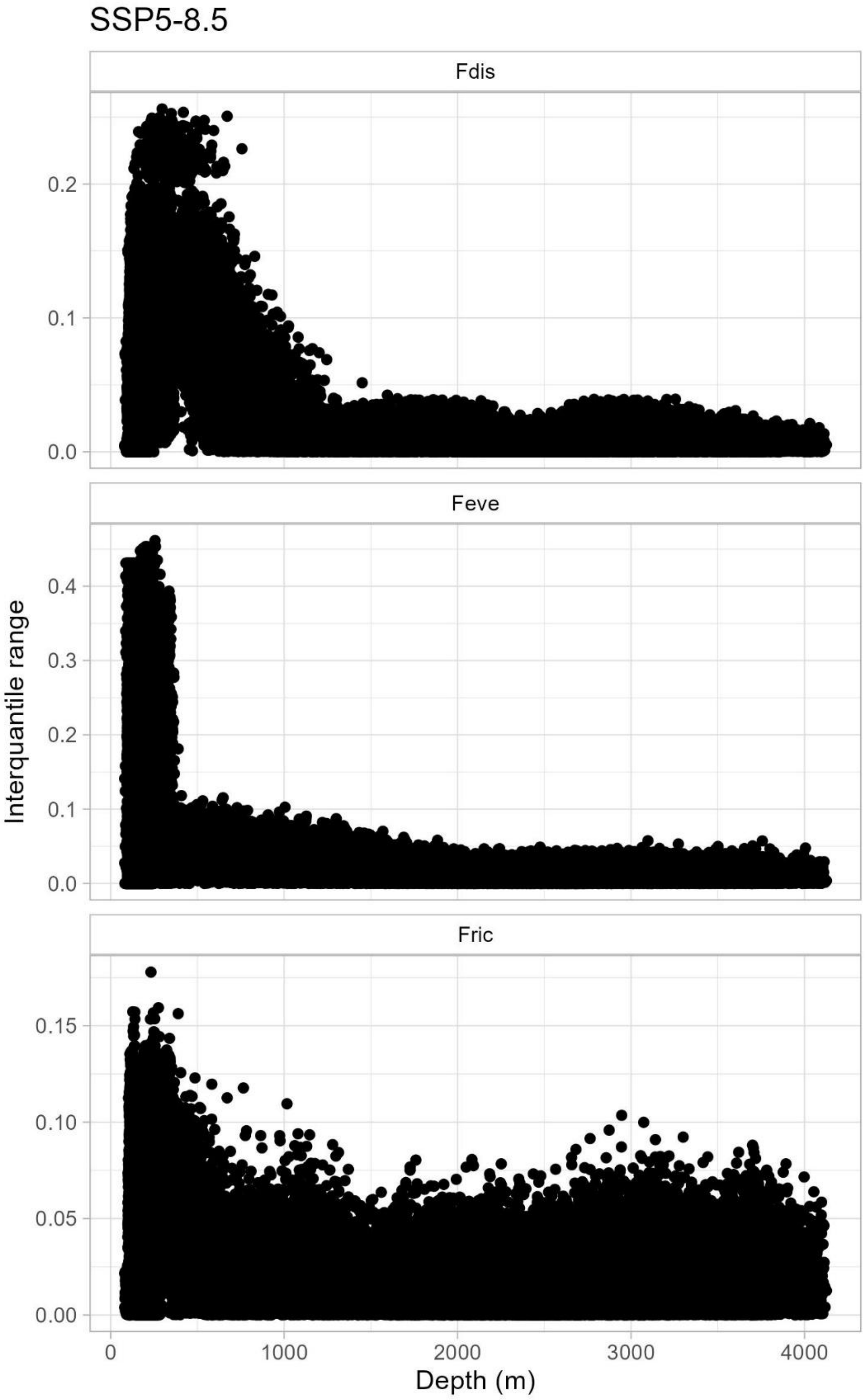

Figure S5.8*:* Interquantile range (range between the 25th and 75th percentile) versus depth for predictions of delta values of three trait diversity indices characterizing deep-water coral communities under the SSP5-8.5. climate scenario. Delta values represent differences between present and future conditions. Fric: Functional richness, Feve: Functional evenness, Fdis: Functional dispersion

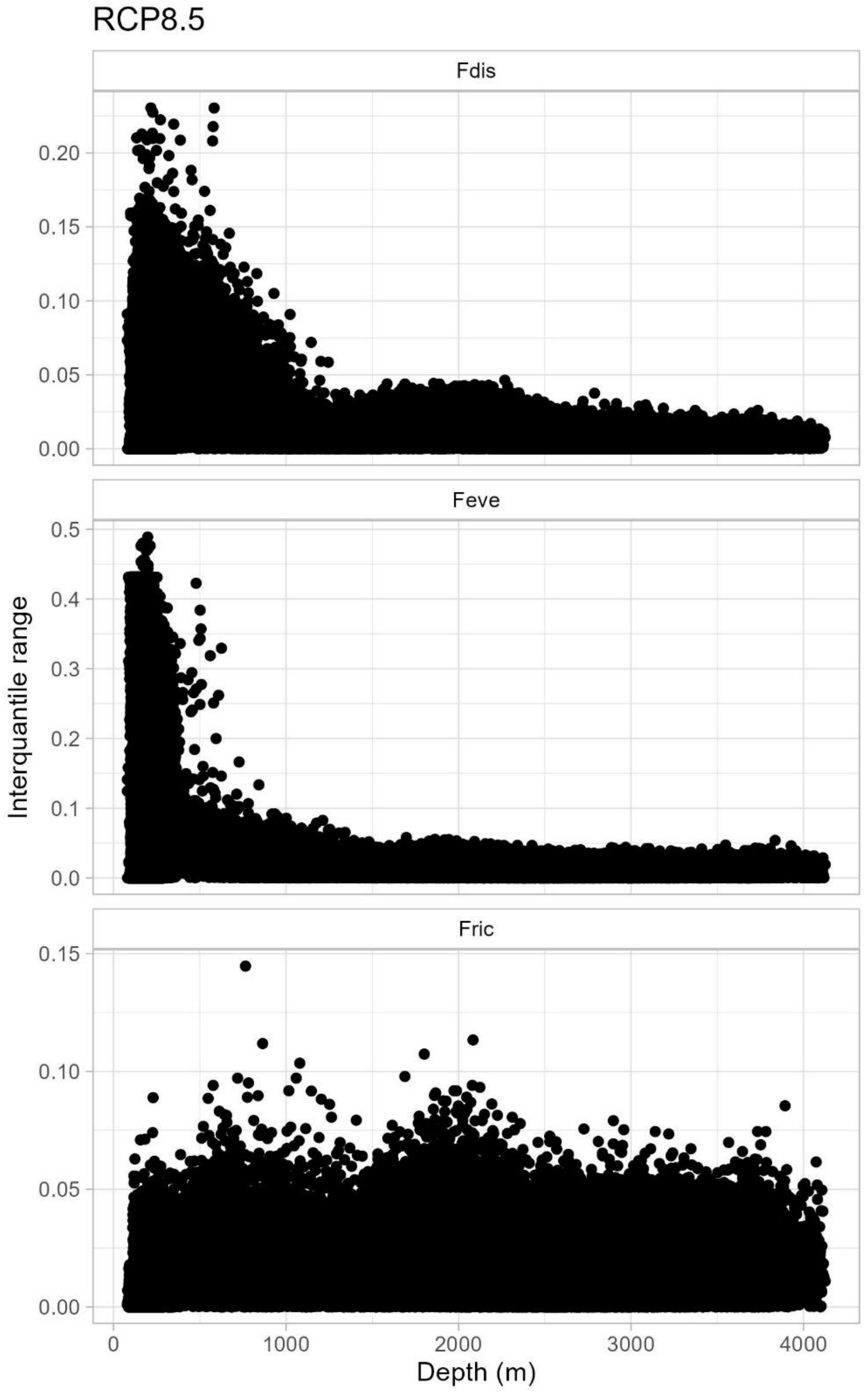

Figure S5.9: Interquantile range (range between the 25th and 75th percentile) versus depth for predictions of delta values of three trait diversity indices characterizing deep-water coral communities under the RCP8.5 climate scenario. Delta values represent differences between present and future conditions. Fric: Functional richness, Feve: Functional evenness, Fdis: Functional dispersion

Community weighted mean

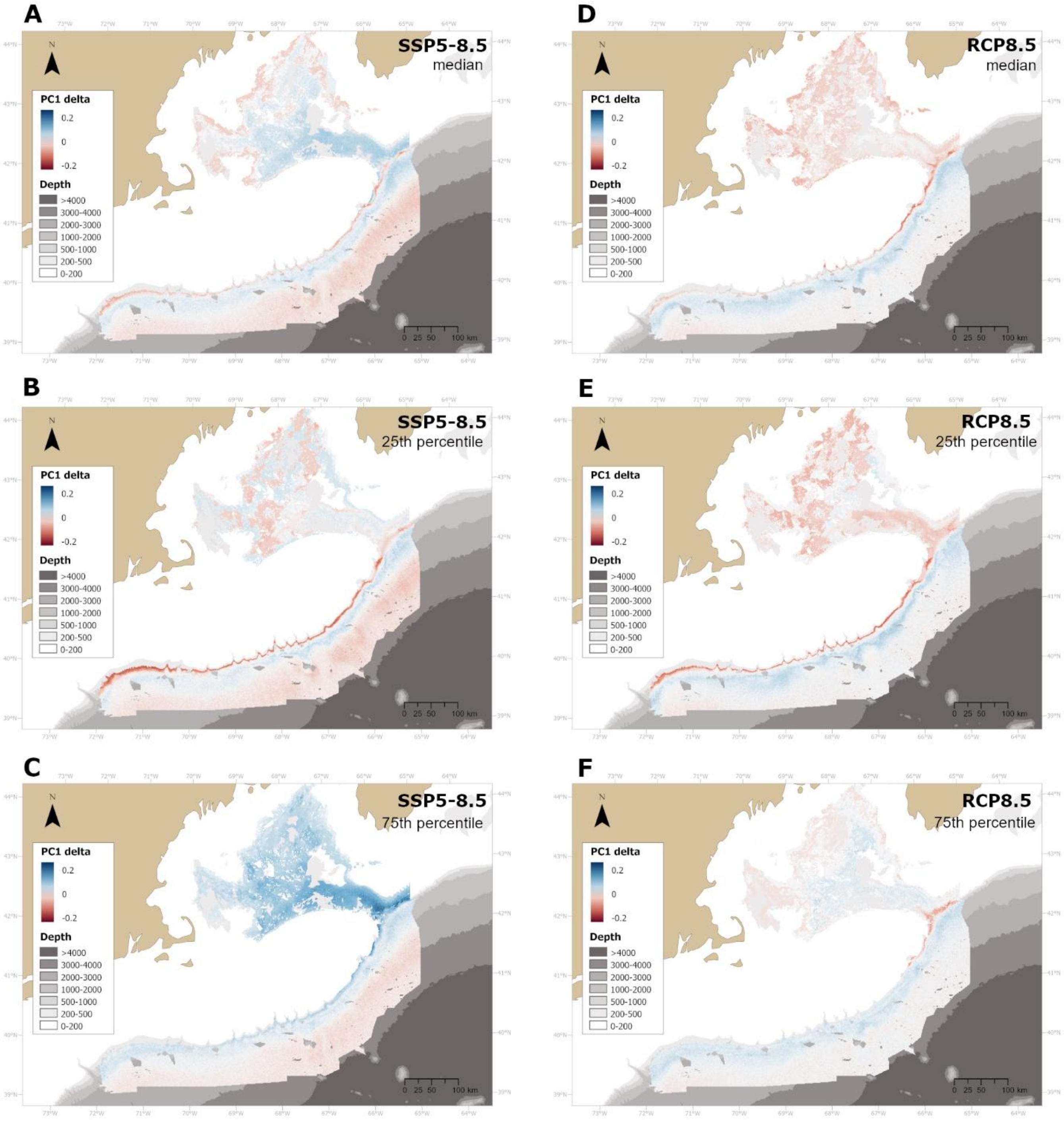

Figure S5.10: Delta values of the community weighted mean of trait axis PC1 (calcite-aragonite axis) characterizing deep-water coral communities under the SSP5-8.5 (A, B, C) and RCP5.8 (D, E, F) climate scenarios. Delta values represent differences between present and future conditions *under the respective*

projection. Top figures (A, D) represent the median, middle figures (B,E) the 25th percentile, and bottom figures the 75th percentile.

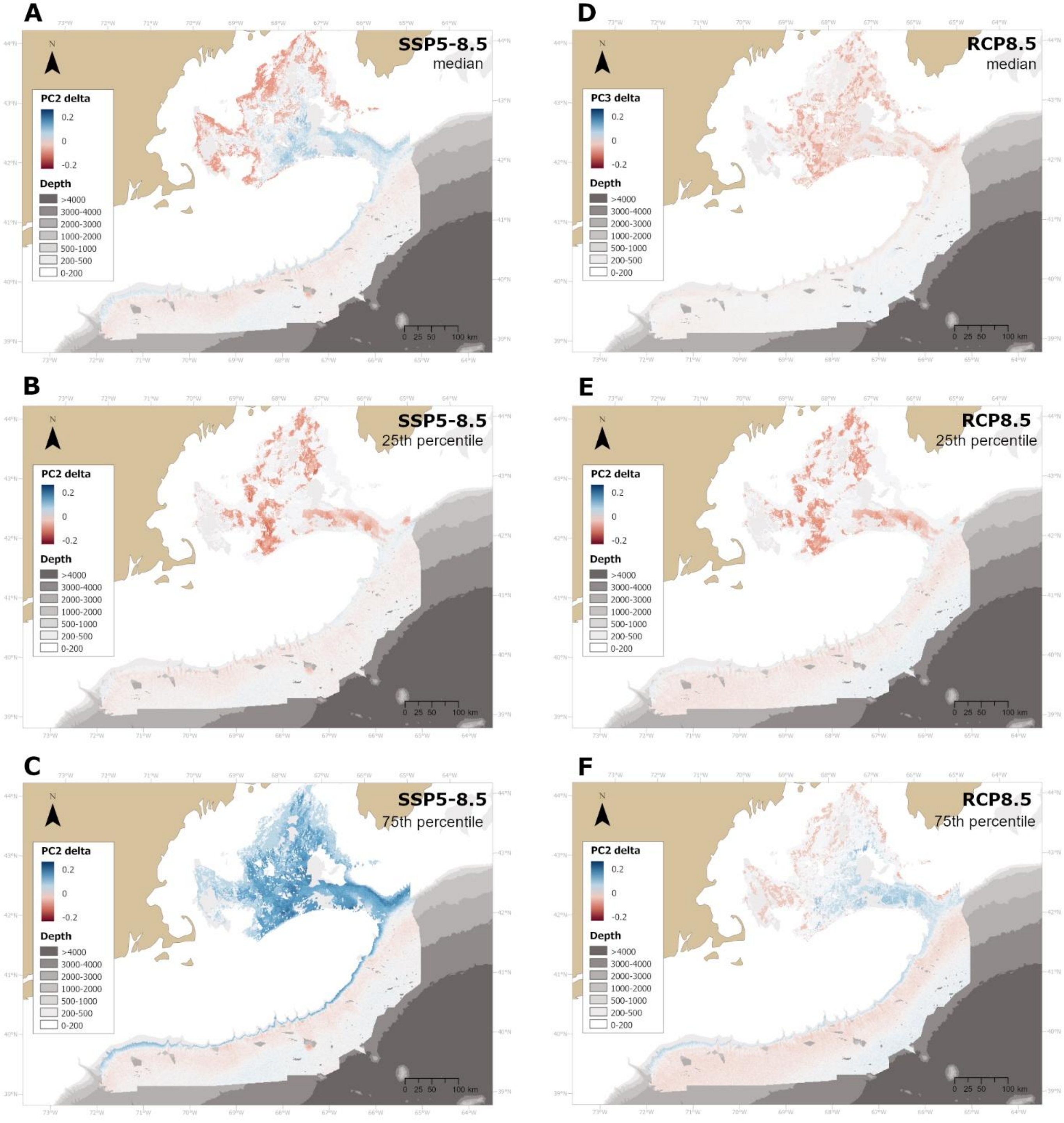

Figure S5.11: Delta values of the community weighted mean of trait axis PC2 (calcite-scleroprotein axis) characterizing deep-water coral communities under the SSP5-8.5 (A, B, C) and RCP5.8 (D, E, F) climate scenarios. Delta values represent differences between present and future conditions *under the respective*

projection. Top figures (A, D) represent the median, middle figures (B,E) the 25th percentile, and bottom figures the 75th percentile.

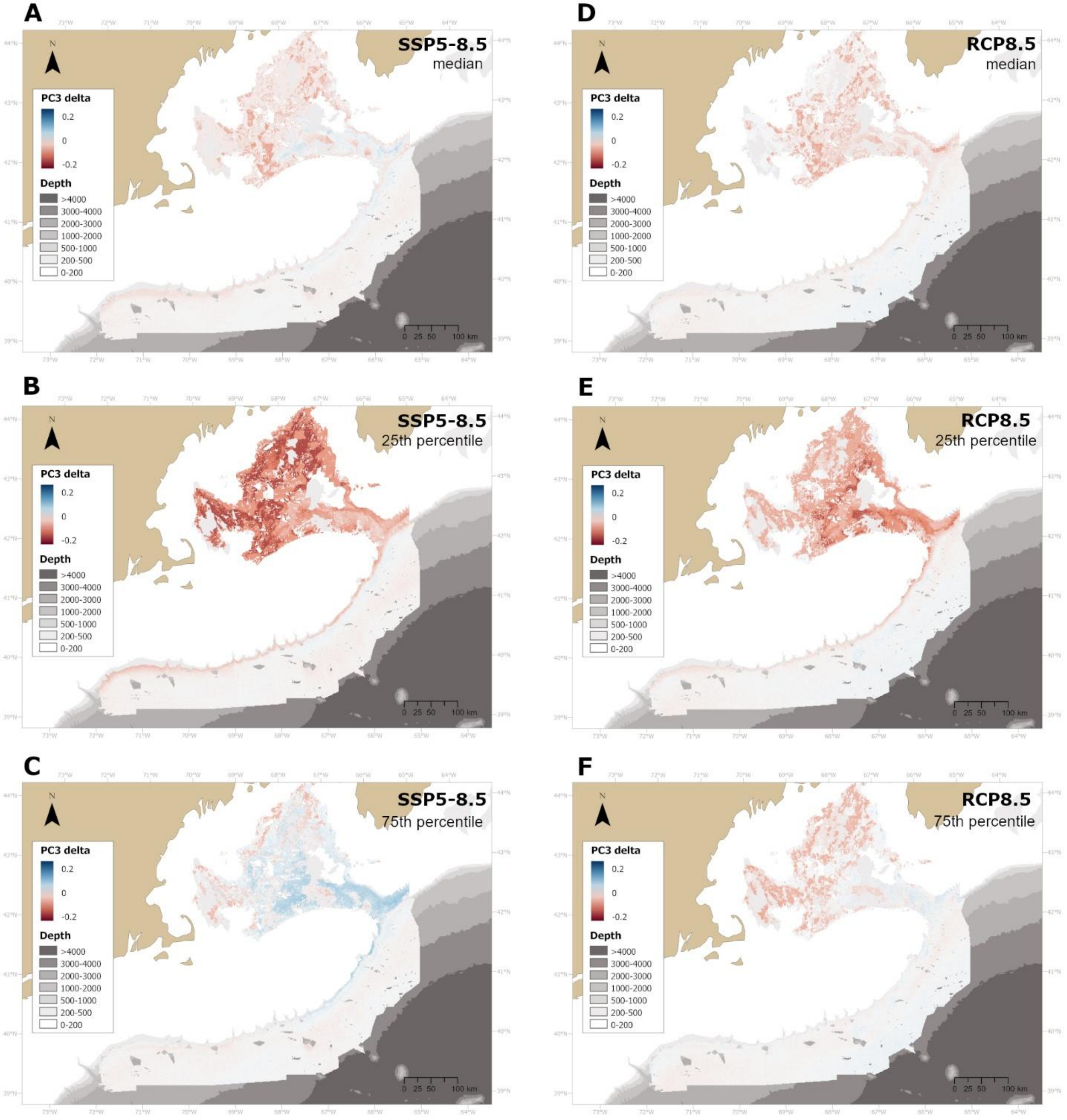

Figure S5.12: Delta values of the community weighted mean of trait axis PC3 (inverted colony size axis) characterizing deep-water coral communities under the SSP5-8.5 (A, B, C) and RCP5.8 (D, E, F) climate scenarios. Delta values represent differences between present and future conditions *under the respective* projection. Top figures (A, D) represent the median, middle figures (B,E) the 25th percentile, and bottom figures the 75th percentile.

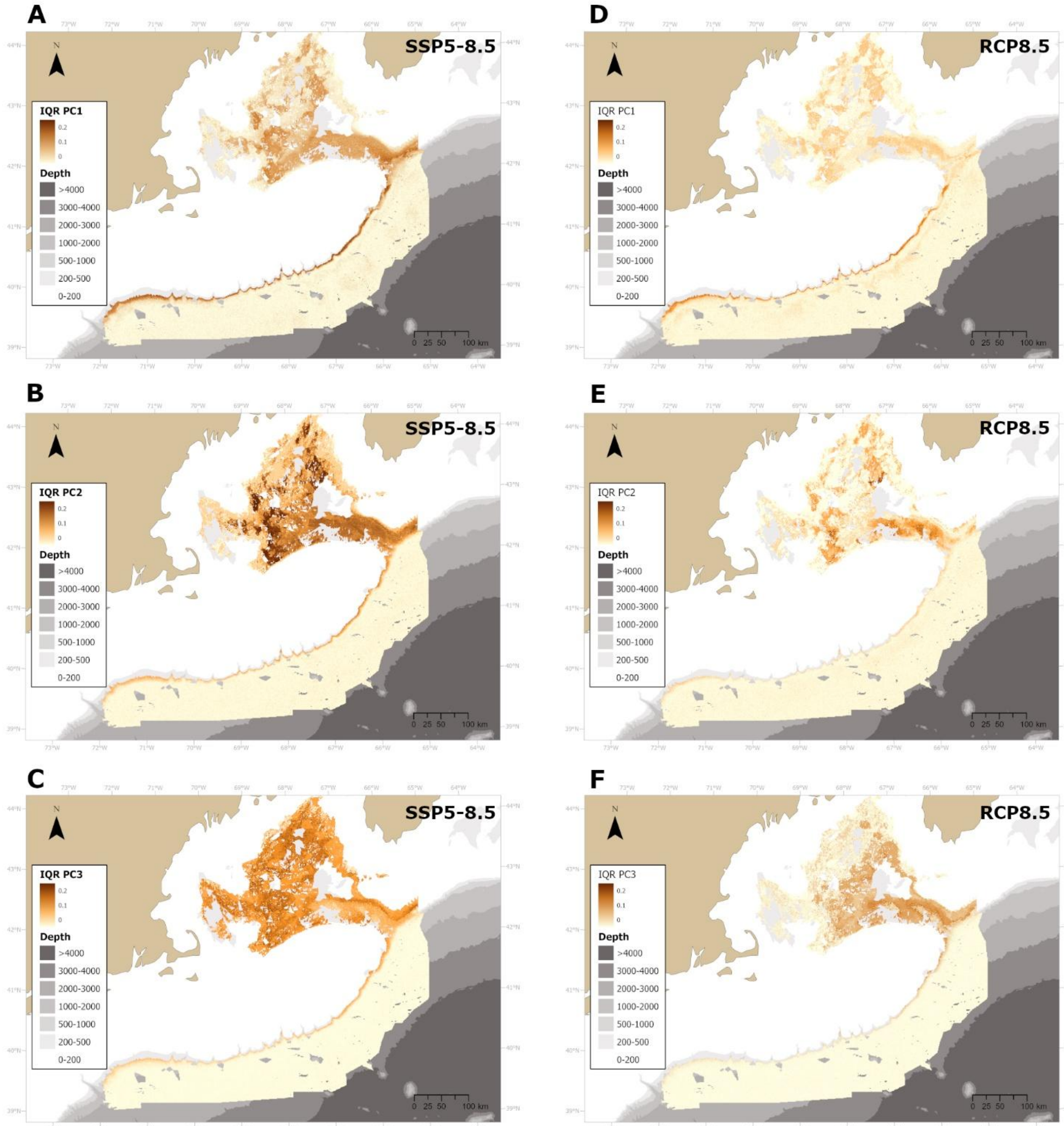

Figure S5.13: Interquantile range (range between the 25th and 75th percentile) for predictions of delta values of the community weighted mean of three trait axes (PC1, PC2, PC3) characterizing deep-water coral communities under the SSP5-8.5 (A, B, C) and RCP5.8 (D, E, F) climate scenarios. Delta values represent differences between present and future conditions under the respective projection. Fric: Functional richness, Feve: Functional evenness, Fdis: Functional dispersion

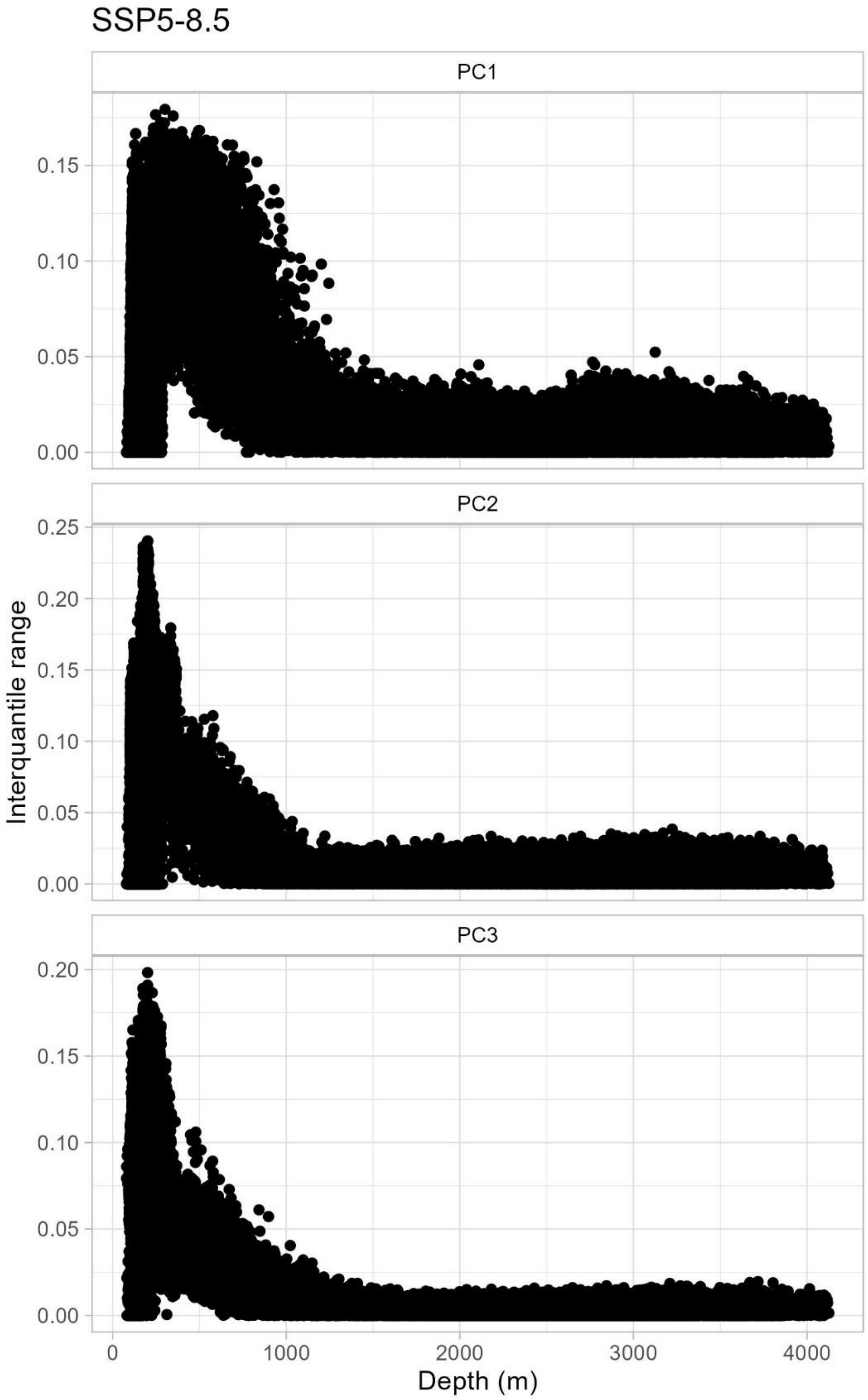

Figure S5.14*:* Interquantile range (range between the 25th and 75th percentile) versus depth for predictions of the community weighted mean of three trait axes (PC1, PC2, PC3) characterizing deep-water coral communities under the SSP5-8.5 climate scenario. Delta values represent differences between present and future conditions.

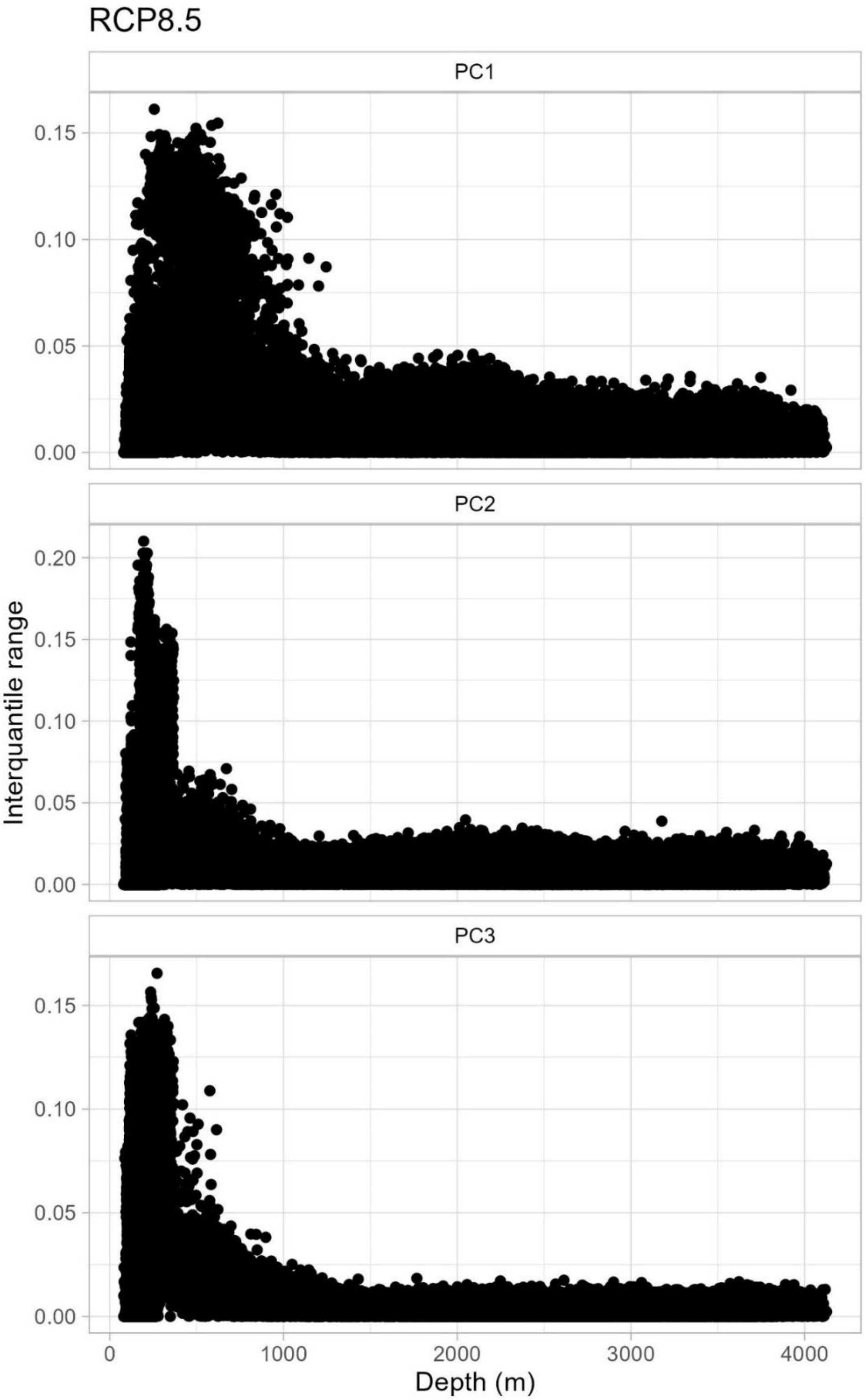

Figure S5.15: Interquantile range (range between the 25th and 75th percentile) versus depth for predictions of the community weighted mean of three trait axes (PC1, PC2, PC3) characterizing deep-water coral communities under the RCP8.5 climate scenario. Delta values represent differences between present and future conditions.
