# Supplementary material for: Climate Change Drives Bathymetric Shifts in Taxonomic and Trait Diversity of Deep‐Sea Benthic Communities
Source: Glob Chang Biol. 2025 Aug 5;31(8):e70407. doi: 10.1111/gcb.70407 (PMC12322877; doi:10.1111/gcb.70407)
Supplement: Supplementary file 6 — Data S6: gcb70407‐sup‐0006‐Supinfo6.pdf. [file GCB-31-e70407-s004.pdf]

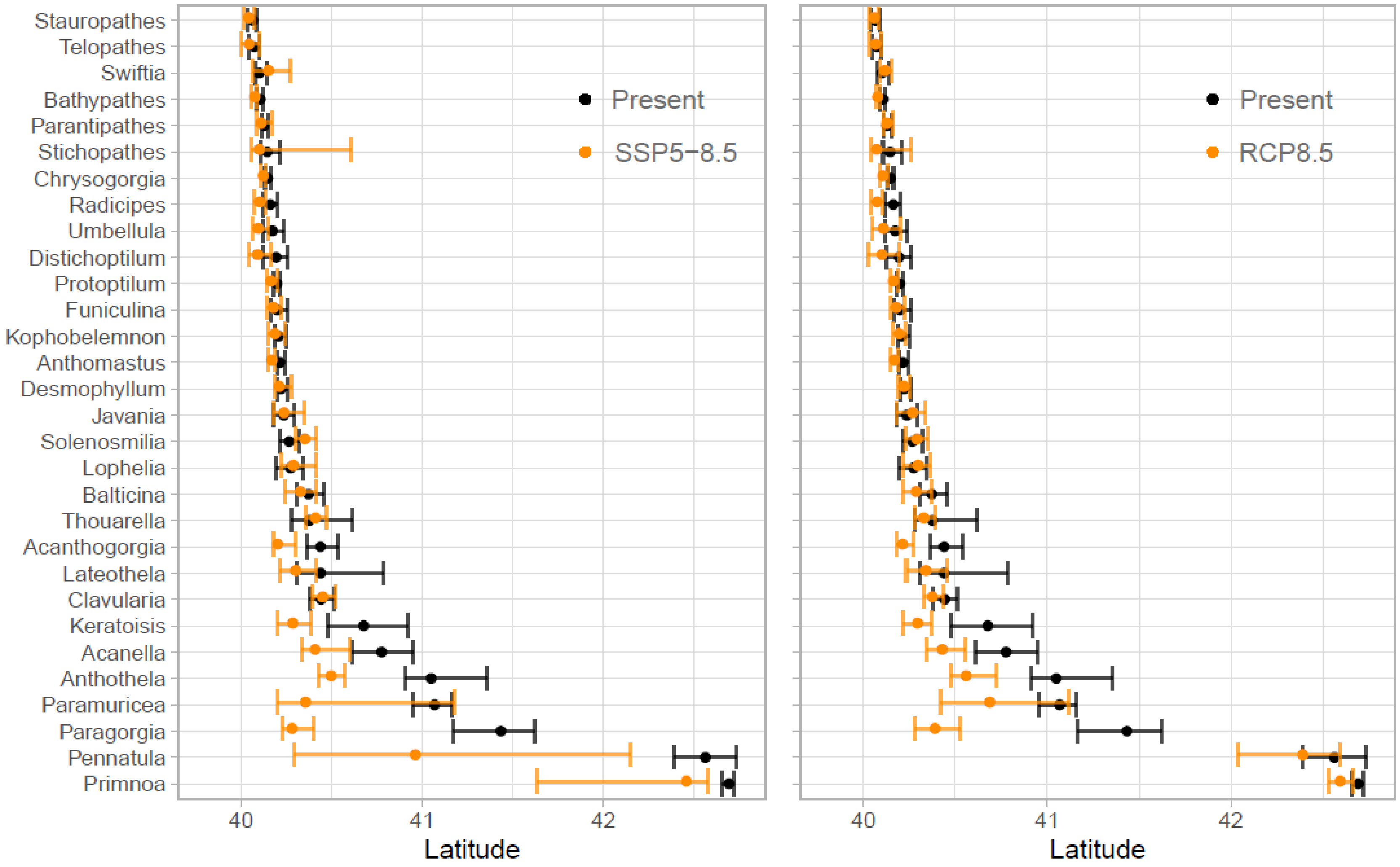

Figure S6.1: Latitude of the centroid of the distribution of 30 deep-water coral genera throughout the study area under present conditions (black colour) and two climate change projections (SSP5-8.5 and RCP8.5, orange colour) for 2100. Points represent the median, while lines represent the interquantile range between the 25th and 75th percentile calculated from 300 predicted datasets for each projection.
